# An air-electrode with hierarchically continuous pore architecture: a step toward “true” lithium–air batteries working with atmospheric oxygen

**DOI:** 10.1080/14686996.2026.2658329

**Published:** 2026-05-08

**Authors:** Akihiro Nomura, Kimihiko Ito

**Affiliations:** Research Center for Energy and Environmental Materials, National Institute for Materials Science, Tsukuba, Japan

**Keywords:** Lithium–air battery, air-electrode, hierarchical pore architecture, carbon nanotube, carbon paper, gas diffusion layer, high energy density, high power density, oxygen reduction reaction

## Abstract

Lithium – air batteries (LABs) are a technology beyond lithium-ion batteries that have high energy density, but they can only operate in high-O_2_ atmosphere because of their low power output capability. The localized oxygen reduction reaction (ORR) clogs the porous air-electrode, prematurely stopping power generation in an air atmosphere that is ~21% O_2_. Here, we have developed a carbon nanotube (CNT)-based air-electrode combined with a carbon paper (CP) gas diffusion layer (GDL), denoted as CNT-with-CP. X-ray computed tomography (XCT) and mercury porosimetry reveal a hierarchical pore architecture between the CNT/CP layers. This architecture has a continuous pore distribution between the nanopores of the CNT layer and micrometer-sized CP voids, which is artificially supported inside the high porosity CP. This pore structure allows continuous O_2_ inhalation without the air-electrode pores being clogged, facilitating uniform ORR across the air-electrode under low-O_2_ gas atmosphere. This enables a fast discharge under an atmospheric O_2_ environment and extends the cycle life of LAB cells. Multiple stacks of CNT-with-CP air-electrodes and lithium foil anodes produced a lightweight Ah-class LAB with high energy density that operates under atmospheric O_2_. This battery had a discharge capacity of 1.6 Ah at a current of 0.10 A per a 5.2 g device, corresponding to an energy density of 740 Wh kg^−1^ at a power density of 48 W kg^−1^. This is the first study demonstrating a step toward ‘true’ LAB working with atmospheric O_2_ to provide a feasible power output in ambient air.

## IMPACT STATEMENT

This study developed a hierarchically continuous porous air-breathing cathode and used it to produce a “true” lithium–air battery that successfully works with atmospheric oxygen.

## GRAPHICAL ABSTRACT

**Figure:**
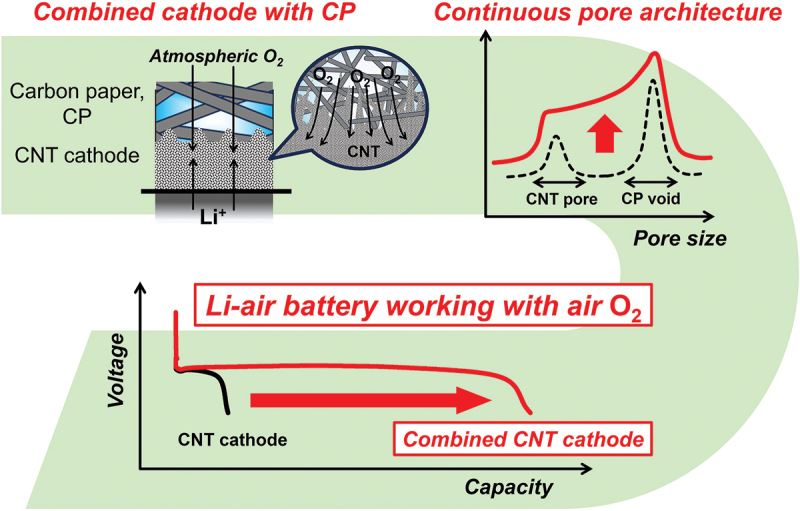

## 1. Introduction

Increasing demand for long-distance mobilities such as electric vehicles (EVs) and electric vertical take-off and landing (eVTOL) aircraft is propelling research and development of batteries beyond the lithium-ion battery (LiB) that have high energy density. This trend is focusing on the lithium – air battery (LAB), which provides electric energy through aerobic oxidation of lithium (2Li + O_2_ ↔ Li_2_O_2_) with a theoretical energy density of 3500 Wh kg^−1^, the highest among any possible rechargeable batteries [1]. Recent progress on LAB materials and cell-assembling technology has realized LAB cells with very high energy densities of >500 Wh kg^−1^ [2–5], demonstrating ultra-high cell capacities with lightweight cell configurations. However, such pioneering works reveal the poor power capability of LABs, which have power densities of no more than 100 W kg^−1^ in a pure O_2_ gas environment. Although this is often recognized as LAB technology, the slow oxidation kinetics requires a concentrated O_2_ gas stream for the cells to work [6–9], so this battery technology is not strictly ‘lithium – air’ (Li – air), but is ‘lithium – oxygen’ (Li – O_2_) more precisely. Li – O_2_ technology is expected to be implemented in a hermetically sealed O_2_ gas cylinder, but realizing battery systems that have high energy density with auxiliary equipments such as gas regulators and piping, is questionable [10,11]. Therefore, apart from the conventional Li-O_2_ battery system, there is an urgent need for a new battery system that operates using lithium metal and low O_2_ gas stream at room temperature. Specifically, to realize practical power sources with high energy density, the system needs to work in atmospheric air containing ~21% O_2_ and ~78% N_2_, as well as traces of Ar, CO_2_ and H_2_O. This is defined as ‘true’ LAB here, but studies on the battery performance under ambient air conditions have rarely been conducted due to the difficulty of deriving affordable power output with the atmospheric O_2_ gas concentration. As a step toward realizing ‘true’ LAB, this study introduces an air-electrode (air-breathing cathode for air-batteries) design to conquer the challenge.

The partial pressure of O_2_ of atmospheric air is one-fifth the pressure of a pure O_2_, which makes a true LAB highly challenging to produce. True LAB cells that discharge and charge under atmospheric O_2_ have been demonstrated with an aqueous catholyte, which has a much faster oxygen reduction reaction (ORR) than that in an aprotic system [12–14]. However, it is a major challenge to achieve high energy density by assembling a battery cell in such an aqueous system, because it is a bi-electrolyte system (consisting of an aqueous catholyte and non-aqueous anolyte) and heavy battery materials (catalysts and electrolytes) need to be loaded to configure the cell structure. In addition, there is a serious safety concern stemming from the accidental contact of the aqueous catholyte with the lithium anode. LAB cells with high energy density (>500 Wh kg^−1^) have been demonstrated mostly in an aprotic system using a single organic electrolyte [2–5], but the significantly slow ORR (2Li^+^ + O_2_ +2e^−^ ↔ Li_2_O_2_) makes the true LAB fatally inactive and confines the system viability only to a high-O_2_ atmosphere (Li – O_2_). Activating a true LAB requires accelerating the ORR on the air-electrode, so much effort has been made to increase the ORR rate of LABs. One way to do this is to incorporate ORR catalysts and/or mediators onto the electrolyte/electrode interface [15–18], but this suffers from the effects of high-mass loading and increasing cell mass. Increasing O_2_ solubility and transport of dissolved O_2_ is another strategy to enhance the ORR rate of an LAB. Perfluorocarbons as electrolyte solvents increase O_2_ solubility and thus raise the ORR rate [19–21]. Low-viscosity amide-based electrolytes enable fast discharge through fast transport of Li^+^ and O_2_ reactants [22–24]. The enhanced oxidative stability of amide molecules also extends the cycle life of amide-based electrolyte cells compared with that of cells with a tetraethyleneglycol dimethylether (TEG)-based electrolyte, which is currently a standard LAB electrolyte. Nevertheless, the effects remain limited to a Li – O_2_ system, and achieving a true LAB that works with atmospheric O_2_ continues to pose a challenge.

Besides the electrolyte/electrode interface, the microporous structure of air-electrodes plays a crucial role in promoting an ORR. Because the exchange current density of an ORR at a carbon electrode surface is 10^−7^ times lower than that of Li dissolution/plating [25], electrodes with high surface area are imperative for generating high currents. To promote the ORR and derive a high capacity, nanocarbon materials with high specific surface area, such as carbon blacks [26,27], carbon nanotubes (CNTs) [28–30], carbon nanofiber [31,32], and graphenes [33–35], have been investigated as air-electrode materials. In addition, air-electrode needs to have a highly porous architecture to facilitate diffusion of the reactants and provide storage space for Li_2_O_2_ discharge products. In principle, a uniform ORR inside the air-electrode helps in achieving a fast discharge [36,37]. A local electrode reaction causes high overpotential and thus limits the current, resulting in early cell death. We have recently demonstrated that a highly porous carbon electrode consisting of non-woven CNT bundles enables a discharge – charge cycle even under atmospheric O_2_ [22]. A thin and lightweight CNT sheet with high porosity of up to 95% significantly enhances the ORR rate, enabling a fast discharge per electrode area of >5 mA cm^−2^ using atmospheric O_2_. This is believed to be due to the high porosity of the CNT electrode, which enhances O_2_ adsorption and promotes a homogeneous ORR within the electrode. However, ORR propagation inside a highly porous carbon air-electrode has been elusive and remains unexplored.

**Figure 1.**
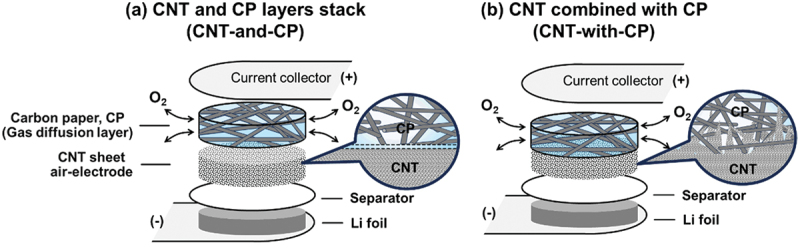
Schematic of stacked LAB cell configurations using (a) CNT-and-CP and (b) CNT-with-CP cathodes.

Here, we investigated the ORR progress during discharge within a highly porous CNT sheet air-electrode. Cross-sectional scanning electron microscopy (SEM) along with observation of the O element distribution via energy-dispersive spectroscopy (EDS) revealed that the ORR is concentrated locally on the gas side of the air-electrode surface, while the separator/Li side remains unreacted. The local ORR blocks the subsequent O_2_ inhalation, shortening the discharge time and resulting in premature cell death. This was more evident in air that was ~21% O_2_, which made the cell discharge under air O_2_ unrealistic. However, this complication can be relieved by forming a hierarchically continuous pore structure on the gas side of the air-electrode. Filtration of CNT slurry on carbon paper (CP), composed of electroconductive carbon fibers (CFs) bonded together in a flat sheet, artificially realizes such hierarchical pore architecture. Instead of simply stacking an individual CNT air-electrode layer and a CP as a gas diffusion layer (GDL) (configuration CNT-and-CP in Figure 1(a)), integrating the CNT sheet electrode with the CP (configuration CNT-with-CP in Figure 1(b)) mitigates the local ORR during discharge and secures the ventilation pathway throughout the discharge process. The latter configuration derives a cell capacity as high as that in 100% O_2_ (Li – O_2_) even under atmospheric O_2_ (Li – air). X-ray computed tomography (XCT) and mercury intrusion porosimetry revealed that the integrated CNT-with-CP cathode creates a continuous pore structure ranging from voids on the scale of a few hundred micrometers to nanometer-scale pores when a high porosity CP was employed, which serve as effective O_2_ vessels to promote a homogeneous ORR inside the whole air-electrode. Multiple stacking of Li/separator/CNT-with-CP layers realized an LAB that passively uses atmospheric O_2_. A 5.2 g LAB device achieved an energy of 3.9 Wh (energy density of 740 Wh kg^−1^) at a 0.10 A current (power density of 48 W kg^−1^) using atmospheric O_2_, which surpasses any existing battery devices, such as lithium-polymer (Li-Po), alkaline, and lithium batteries in energy density at practical power conditions. This finding reveals how the air-electrode should ideally be designed to obtain a true LAB that works with atmospheric O_2_.

## 2. Experiments

### 2.1. Cell assembly in CNT-and-CP configuration

Single-walled CNTs (ZEONANO® SG101, obtained from Sigma-Aldrich, Japan) were dispersed in deionized water at a concentration of 0.1 wt% using an ultrasonic homogenizer (Branson 450D, Emerson, USA). The slurry was then filtered under vacuum through a PTFE membrane with a pore size of 1 μm (Omnipore™, EMD Millipore, obtained from Sigma-Aldrich, Japan) to obtain a free-standing binder-free CNT sheet. The filtration amount was adjusted to obtain a 130 μm thick CNT sheet with a CNT loading of 2.1 mg cm^−2^. After drying in a vacuum overnight, the CNT sheet was peeled off the membrane and cut into a circle (16 mm in diameter, electrode area of 2 cm^2^). A stacked LAB cell was fabricated by layering a 16 mm diameter lithium foil (200 μm thick, Honjo Metal, Japan), a 19.5 mm diameter porous polyolefin separator, a CNT sheet electrode, and a 16 mm diameter CP as a GDL, between the two current collectors to form the CNT-and-CP configuration. The CNT layer and separator were impregnated with 24 μL (12 μL cm^−2^ per electrode area) of electrolyte composed of TEG (Japan Advanced Chemicals) dissolving 1.0 M of lithium bis(trifluoromethanesulfonyl)imide (LiTFSI) (Kishida Chemical Co., Ltd., Japan), denoted as TEG-TFSI. The stack was fixed with spring clamps under a pressure of 118 kPa.

### 2.2. Cell assembly in CNT-with-CP configuration

CNT air-electrode combined with CP (CNT-with-CP) was prepared by directly filtering a CNT slurry through a CP to line the CP with a CNT loading of 2.1 mg cm^−2^. Through the filtration process, a part of the CNT component of the CNT layer digs into the void of CP, so the two layers are stuck together to provide a CNT sheet air-electrode supported on CP. A stacked LAB cell was fabricated in the same way, by layering a lithium foil, a separator, and a CNT-with-CP cathode between the current collectors to form the CNT-with-CP configuration. This cell configuration was impregnated with 24 μL (12 μL cm^−2^ per electrode area) of TEG-TFSI electrolyte and fixed in the same manner as the CNT-and-CP configuration.

### 2.3. Battery testing and characterization

Galvanostatic discharging and charging were conducted using a battery tester (HJ1001SD8, Hokuto Denko, Japan) at room temperature under a pure O_2_ gas stream (>99.8% O_2_, <1 ppm CO_2_, <1 ppm H_2_O) or in a dry room with an environmental dew point of approximately −60 to −50°C (10–40 ppm H_2_O) and the environmental O_2_ and CO_2_ gas concentrations of ~21% and <400 ppm, respectively. The discharge and charge cutoff voltages were set to 2.0 V and 4.5 V, respectively. The cell discharge energy was obtained by integrating the discharge curve area (V-mAh) until the cutoff voltage of 2.0 V. Cell power is defined as the average discharge power obtained by dividing the discharge energy by the discharge time. CNT air-electrode morphologies were analyzed using a field emission scanning electron microscope (FE-SEM) (JSM-7800F, JEOL, Japan) with an accelerating voltage of 5 keV. The element distribution was analyzed using an energy-dispersive spectroscopy (EDS) analyzer (X-Max^N^, Oxford Instruments, UK) equipped with the SEM. After the discharge experiments, the air-electrodes were taken out from the disassembled cells in the dry room, thoroughly rinsed with super-dehydrated acetonitrile (<10 ppm H_2_O, Fujifilm-Wako, Japan) and then dried under vacuum to be prepared for observation. The Brunauer – Emmet – Teller (BET) surface area was determined from the nitrogen adsorption isotherm at 77 K (3FLEX, Micromeritics, USA). The pore size distribution for nanometer-scale pores was evaluated using the Barrett–Joyner–Halenda (BJH) model. A mercury intrusion porosimeter (AutoPore IV 9500, Micromeritics, USA) was used to determine the size distribution of micrometer-scale pores. XCT analyses of the air-electrode line with CP were carried out using an Xradia 520 Versa (ZEISS, Germany) instrument, where the source voltage and power were 80 kV and 7 W, respectively. The XCT imaging resolution was 1.97 μm. X-ray diffraction (XRD) spectra of CNT air-electrodes were recorded by an X-ray diffractometer (SmartLab, Rigaku, Japan) using a CuKα source (λCuKα = 1.542 Å). Cell weight change during discharge and charge was monitored using a homemade gravimetric analysis system described in the literature [38]. Online differential electrochemical mass spectroscopy (DEMS) was performed on the LAB cells using a quadrupole mass spectrometer (JMS-Q1500, JEOL, Japan) with flowing He as the carrier gas to monitor the gas evolution during charging.

## 3. Results and discussion

### 3.1. ORR progress during discharge

**Figure 2.**
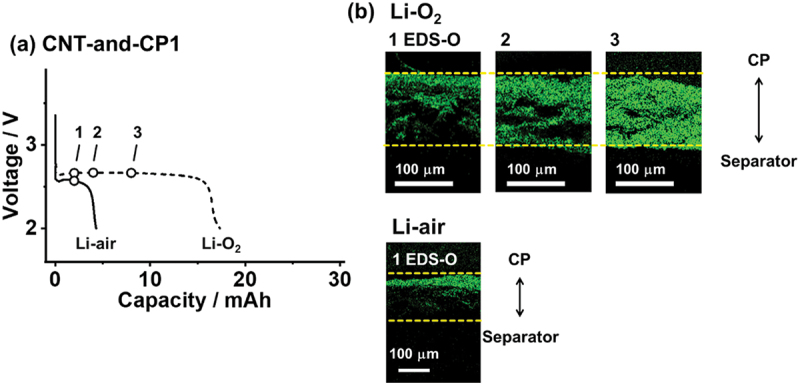
(a) Discharge curves of CNT-and-CP1 cells under pure O_2_ (Li – O_2_, dashed line) and dry air (Li – air, solid line) at a current of 0.8 mA. (b) EDS elemental O mappings of CNT layer cross sections at points 1, 2, and 3 in (a), showing the Li_2_O_2_ discharge product distribution inside the CNT cathode after 2, 4, and 8 mAh discharges, respectively. The image contrast is adjusted to differentiate the element distribution. The dashed yellow lines indicate the bottom (separator side) and top (CP gas side) surfaces of the CNT layer. Additional SEM images and EDS spectra are shown in figures S5 and S6.

To observe the ORR progress inside the air-electrode, LAB stack cells (Figure 1) were fabricated. The cell materials were sandwiched between non-gas permeable metal plates as current collectors, so the cells discharge and charge by O_2_ gas exchange through the CP cross section. The stack cell configuration allows multiple stacking of the air-electrodes with a secure O_2_ inhalation pathway to the electrodes [39,40], enabling us to assemble LAB cells with high energy density and Ah-class capacity, as discussed later. Three commercial CPs (CP1, 2 and 3) were tested in this study and their characteristics are tabulated in Table S1, their SEM images are in Figure S1, and their pore size distribution is in Figure S2. For the ORR progress observation, 300 μm thick CP with the highest porosity (0.94) and largest void width (130 μm), designated CP1 (E704, Kureha, Japan), was used. The ORR on electroconductive carbon fibers (CFs) composing CPs can be ignored because their surface area is negligible compared with that of a CNT [41]. The SEM images of the CNT sheet prepared by CNT slurry filtration are in Figure S3, showing the non-woven textile of aggregated CNT bundles with an inter-bundle gap of 10^−1^–10^1^ μm. The N_2_ adsorption isotherm (Figure S4) reveals the presence of miniscule pores with a size of <50 nm inside the aggregated CNT bundles, providing a large BET surface area of 870 m^2^ g^−1^. The pore size distribution and surface area were not significantly changed by the filtration bases (PTFE filtration membrane or CP) used to form the CNT layers. However, the filtration bases considerably alter the pore structure that is out of the detection range of N_2_ adsorption, as discussed later. First, the free-standing CNT sheet obtained by filtration on the PTFE membrane was stacked with CP1 (300 μm thick) to assemble a stack cell (CNT-and-CP1) and observe the ORR progress. The amount of electrolyte (24 μL) corresponds to the void volume of the CNT cathode and separator layers, ensuring they are fully wetted without overflowing. Figure 2(a) shows the discharge curves of the cells tested under an O_2_ gas flow (Li – O_2_) and dry air (Li – air). In Li – O_2_, a stable voltage plateau of 2.7 V with a capacity of 17.3 mAh was achieved. In contrast, in Li – air, the capacity significantly decreased to 4.3 mAh, indicating the difficulty of deriving sufficient battery energy under atmospheric O_2_.

The ORR progress was observed via cross-sectional SEM observations of the CNT air-electrodes after 2, 4, and 8 mAh discharges. Figure 2(b) shows the EDS elemental O distribution of the cross section of these air-electrodes after discharge (SEM images of the sheet surfaces and EDS spectra are shown in Figures S5 and S6). Because the discharge product of Li_2_O_2_ deposits on the CNT surface, the elemental O distribution visualizes the extent of ORR progress: higher O signal intensity indicates the part where the ORR lasted longer [31]. The shift in O intensity with increasing capacity reveals a concentrated ORR on the gas side (upper surface, CP side) that was in contact with CP1. In Li–O_2_, the O intensity was higher on the upper surface of the air-electrode than on the separator side (Figure S5). As discharge proceeded, O_2_ infiltrated the separator side (lower surface) to drive the reaction forward. Such localized reactions on the gas side surface have been reported in various papers [4,21,31], indicating that the O_2_ supply, rather than Li^+^ diffusion, limits the LAB capacity and current. Despite the fact that the O_2_ molecule is larger than Li^+^, the diffusion coefficient of O_2_ (*D*_O2_) is 10^1^–10^2^ times higher than that of Li^+^ (*D*_Li+_) in various aprotic electrolytes [42]. This is due to the solvation of Li^+^ or undissociated Li salt in electrolytes, which forms Li^+^-coordinated molecules that are larger than O_2_. Although O_2_ tends to diffuse, it is much less soluble in aprotic electrolytes (~10^−4^ M) than Li^+^ (~1 M) [22], which limits the O_2_ supply during the LAB discharge. Furthermore, O_2_ has one-fifth the solubility in Li – air, which poses a more severe problem. In Li – air (Figure S6), the localized ORR on the gas side was found to block the inter-bundle gaps of the gas side surface of the air-electrode at a discharge of only 2 mAh, which stopped the ORR progress and left the bottom surface of the air-electrode (separator side) almost unreacted. For the same output current, the scarce O_2_ in Li – air was entirely consumed on the gas side to locally deposit Li_2_O_2_, which blocked the O_2_ adsorption path, thus leading to early cell death.

### 3.2. A CNT sheet air-electrode combined with CP (CNT-with-CP)

**Figure 3.**
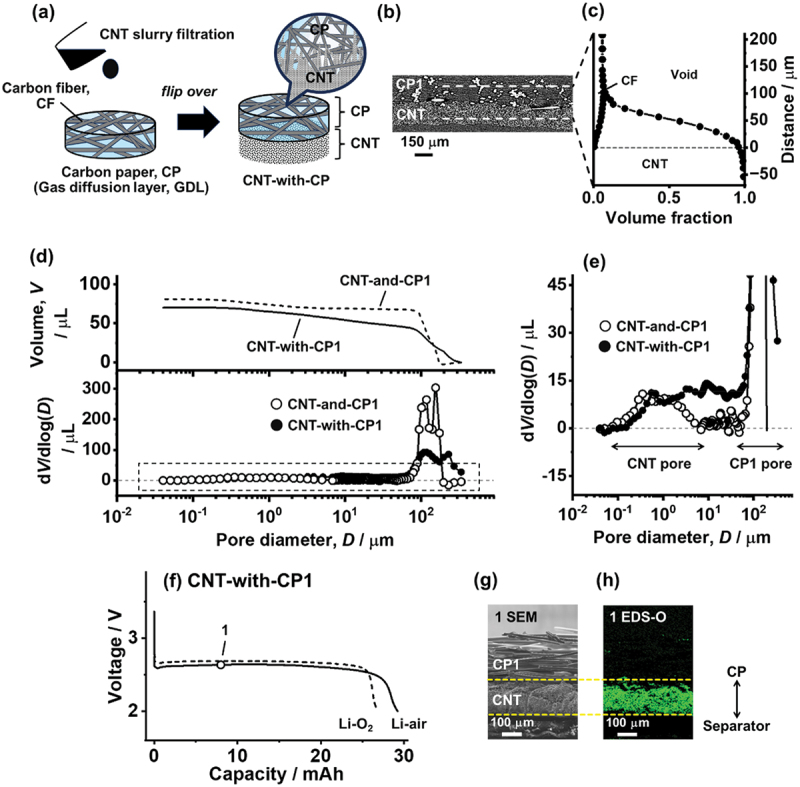
(a) Schematic of the CNT-with-CP preparation. A dispersed CNT slurry was filtrated directly on CP. (b) XCT cross-sectional image of CNT-with-CP1. (c) Volume fractions of CNT, CP void, and CF components near the interface of CNT and CP1. (d) Pore size distribution (down) and cumulative pore volume (up) of CNT-and-CP1 and CNT-with-CP1. (e) Enlarged pore size distribution of the dashed rectangular region in (d). (f) Discharge curves of CNT-with-CP1 cells under pure O_2_ (Li – O_2_) and dry air (Li – air) at a current of 0.8 mA. (g) SEM and (h) EDS elemental O mapping of the CNT-with-CP1 cross section at point 1 in (f), showing the Li_2_O_2_ distribution inside the CNT layer after an 8 mAh discharge under dry air. The dashed yellow lines indicate the bottom (separator side) and top (CP1 side) surface of the CNT layer.

The concentrated ORR at the gas side of air-electrodes clogs the air O_2_ gas inlet to stop the cell discharge. We here introduce an easy approach for preventing electrode clogging and promoting a uniform ORR to derive high capacity in Li–air, by creating a hierarchically continuous pore architecture through the CNT slurry filtration on CP (CNT-with-CP, Figure 3(a)). The cross-sectional SEM image of CNT-with-CP1 (Figure S7) reveals the highly integrated CP1 and CNT layers. To observe the three-dimensional (3D) structure of the integrated layers, an XCT scan was conducted on CNT-with-CP1. Figure 3(b) shows the XCT cross-sectional image of the sheet composite, revealing the CNT component partly penetrates into the void space of CP1 (additional 3D XCT and cross-sectional images of CNT-with-CP1 are shown in Figure S8). Figure 3(c) shows the volume fractions of the CNT component, CP void, and CF near the CNT/CP1 interface plotted against the thickness direction (the spatial component separations are shown in Figure S8). The fractional distribution reveals that the CNT component penetrates into the CP layer up to 100 μm from the outermost surface of CP1 (gray dashed line). This penetration reduces the thickness solely composed of CNT to 80 μm, from the 130 μm thickness for a single free-standing sheet. The CNT layer can be considered to extend the sheet volume by doubling the top 50 μm to 100 μm to form a total CNT layer thickness of 180 μm.

The CNT component expansion into the CP layer creates novel pore architecture for O_2_ inhalation, which can be clarified using mercury intrusion to measure micrometer-scale pores. Figure 3(d,e) shows the pore volumes and pore size distributions of the two cathodes, CNT-and-CP1 and CNT-with-CP1. The measurement for CNT-and-CP1 was conducted by putting a single free-standing CNT sheet and CP1 in a 16 mm diameter dimension into a mercury intrusion cell, while a piece of CNT/CP1 composite (16 mm in diameter) was enclosed in a mercury cell for the CNT-with-CP1 measurement. The CNT-and-CP1 pore distribution simply shows two pore regions distinctly separated by size: 10^−1^ to 10^1^ μm pores for the CNT sheet and 100–200 μm pores for the CP1 void. In contrast, CNT-with-CP1 exhibits a continuous pore structure. Because the CNT penetration into CP1 reduces the CP1 void volume, the total pore volume of 70 μL is slightly lower than that of CNT-and-CP1 (81 μL). The CNT intrusion obscures the two separated pore regions, forming a hierarchically continuous pore architecture with a seamless pore distribution from 10^−1^ to 10^2^ μm. The continuous pore structure develops near the CNT/CP1 interface, and the CNT layer remains 80 μm thick (60% of the free-standing CNT sheet). Despite this integrated pore structure, it is expected that, when 24 μL of electrolyte is injected into a cell, capillary force will confine the electrolyte into the smaller voids of the separator and CNT components rather than the CP voids.

Because of the hierarchically connected pore architecture, CNT-with-CP1 drastically improves the discharge of LAB cells. Figure 3(f) shows the discharge curves of CNT-with-CP1 cells in Li – O_2_ and Li – air. Although the discharge voltage plateau of Li – air (2.6 V) is slightly lower than that of Li – O_2_ (2.7 V), Li – air maintains a stable discharge voltage to achieve a capacity of 29.2 mAh, six times higher than that of CNT-and-CP1 in Li – air (4.7 mAh). The Li – O_2_ cell capacity also improves from 17.3 mAh for CNT-and-CP1 to 26.8 mAh for CNT-with-CP1. This demonstrates that continuous pore structure enhances the discharge regardless of the O_2_ gas concentration. The capacity of approximately 30 mAh (capacity per unit area of 15 mAh cm^−2^ and capacity per unit CNT mass of 7100 mAh g^−1^) is considered to be the maximum discharge capacity of the CNT air-electrode with CNT loading of 2.1 mg cm^−2^. The 30 mAh discharge consumes the 75 μm thick Li foil to deposit nominally 60 μm thick Li_2_O_2_ inside the air-electrode, occupying ~50% of the inter-bundle gap of the CNT layer. We have previously confirmed this maximum capacity (~30 mAh) for the free-standing CNT air-electrode in a cell configuration with forcible pure O_2_ gas injection [23]. Although more discharge capacity can be obtained by allowing CNT sheet swelling during discharge, this collapses the electrochemical integration of the electrode. Therefore, the ~30 mAh discharge is considered to be the practical maximum for the CNT air-electrode. The Li – air capacities approaching to this maximum verify the successful LAB operation under dry air, which is ascribed to the continuous micrometer-scale pores created on the gas side surface (CP side) of the CNT air-electrode. The integrated CNT and CP layers alleviate the ORR concentration on the gas side electrode and thus secure the O_2_ diffusion path during discharge. The cross-sectional SEM and the EDS elemental O measurement of CNT-with-CP1 after an 8 mAh discharge (Figure 3(g,h)) well reveal this behavior, demonstrating uniform ORR progress in the depth direction of the CNT air electrode in Li – air.

**Figure 4.**
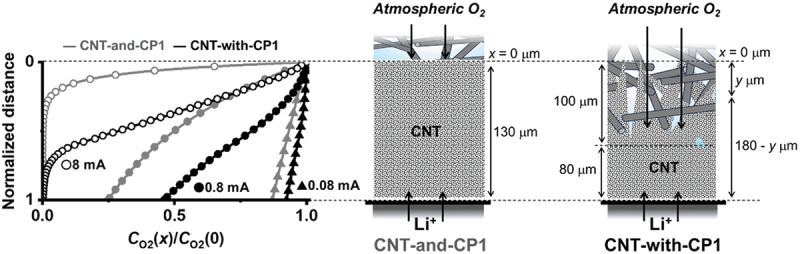
Dimensionless oxygen concentration *C*_O2_(*x*)/*C*_O2_(0) against normalized CNT layer thickness. The grey and black symbols represent the CNT-and-CP1 and CNT-with-CP1 cathodes, respectively.

To understand the O_2_ diffusion behavior, the O_2_ distribution inside a porous electrode was calculated using the model reported by Lu et al. [43]. When the discharge rate is balanced with O_2_ consumption, the O_2_ concentration in the depth direction of the porous air-electrode filled with electrolyte can be expressed as

(1) $$C\text{O}2\left(x\right)=C\text{O}2\left(0\right)\text{exp}\left(-x\frac{j}{\mathrm{nF}C_{\text{O}2}\left(0\right)D_{\text{eff}}}\right),$$

where *C*_O2_(*x*) denotes the O_2_ concentration in the electrode at depth *x* (0–130 μm for a single CNT layer) from the gas/electrode interface, *C*_O2_(0) is the O_2_ solubility in the electrolyte (0.60 mM in 1.0 M TEG-TFSI at 295 K and 1 atm air [42]), *j* is the current per ORR unit surface area, *n* is the number of electrons in the rate-limiting step (assumed to be 1 considering one-electron reduction [25]), *F* is the Faraday constant (96485 C mol^−1^), and *D*_eff_ is the effective diffusion coefficient of O_2_ in the electrolyte inside the porous electrode. *j* was derived by dividing the applied current by the surface area of the CNT air electrode determined by mercury intrusion (0.10 m^2^ per 16 mm diameter sheet) that corresponds to the CNT bundle surface where the ORR dominates. *D*_eff_ is defined as *D*_eff_ = *D*_O2_ε^*β*^, where *D*_O2_ is the diffusion coefficient of O_2_ in 1.0 M TEG-TFSI electrolyte (1.6 × 10^−7^ cm^2^ s^−1^ [42]), ε is the porosity of the air-electrode (0.87), and *β* is the Bruggeman coefficient assumed to be 1.5 [43]. Figure 4 shows the dimensionless O_2_ concentration *C*_O2_(*x*)/*C*_O2_(0) (gray lines) at currents of 0.08, 0.8, and 8 mA plotted against the normalized electrode thickness (the plots against depth *x* are shown in Figure S9). If the reduced O_2_ molecules readily convert into the discharge product Li_2_O_2_, the dimensionless O_2_ concentration *C*_O2_(*x*)/*C*_O2_(0) distribution follows the Li_2_O_2_ distribution, and the accumulated *C*_O2_(*x*)/*C*_O2_(0) area represents the discharge capacity at each current. The calculated *C*_O2_(*x*)/*C*_O2_(0) well describes the inhomogeneous ORR behavior. Although a low current discharge (0.08 mA) ensures a stable discharge via slow but sufficient O_2_ permeation to the bottom surface, a concentrated ORR at the gas surface halves the capacity at the middle current of 0.8 mA and becomes fatally significant and results in negligible capacity at the elevated current of 8 mA. Because the calculation does not consider pore clogging of the electrode by Li_2_O_2_ deposition along with the discharge, the heterogeneity should be more serious in practice. This explains the early cell death of CNT-and-CP1 in Li – air, virtually restricting LAB technology to only low-power devices.

We then calculated the O_2_ distribution for the CNT air-electrode combined with the GDL (CNT-with-CP1). The CNT air-electrode was assumed to be composed of the two pure CNT layers that were 80 μm thick and the 100 μm thick penetration into the CP1 layer (for a total active electrode thickness of 180 μm). The pure-CNT amount of the penetrated layer was assumed to be proportional to the penetrated thickness, so *C*_O2_(*x*) can be expressed as a simple average of *C*_O2_(*x*) throughout a thickness of (180 − *y*) μm, where *y* is the distance inside the penetrated layer from its top surface (0 ≤ *y* ≤ 100):

(2) $$C\text{O}2\left(x,y\right)=\text{​​}C_{\text{O}2}\left(0\right)\left(0\leq x\leq y\right)C_{\text{O}2}\left(0\right)\exp\left(-\left(x-y\right)\frac{j}{\mathrm{nF}C_{\text{O}2}\left(0\right)D_{\text{eff}}}\right)\left(y\leq x\leq 180\right)\text{.}$$

(3) $$C\text{O}2\left(x\right)=\int_{0}^{100}C_{\text{O}2}\left(x,y\right)\mathrm{dy}.$$

The black lines in Figure 4 show the dimensionless O_2_ concentration against the normalized electrode thickness. *C*_O2_(*x*)/*C*_O2_(0) is enhanced because the penetrated layer has more contact with the gas phase. This is more evident at the elevated currents of 0.8 and 8 mA, explaining the enhanced cell capacity by CNT-with-CP1 cathode. The O_2_ diffusion calculation just counts the simple accumulation of electrodes of different thicknesses, so it does not reveal the exact pore size range contributing to the uniform ORR. However, the continuous pore architecture seamlessly connecting the CNT nanoscale pores and CP micrometer-scale voids is responsible for the enhanced O_2_ diffusion across the electrode.

**Figure 5.**
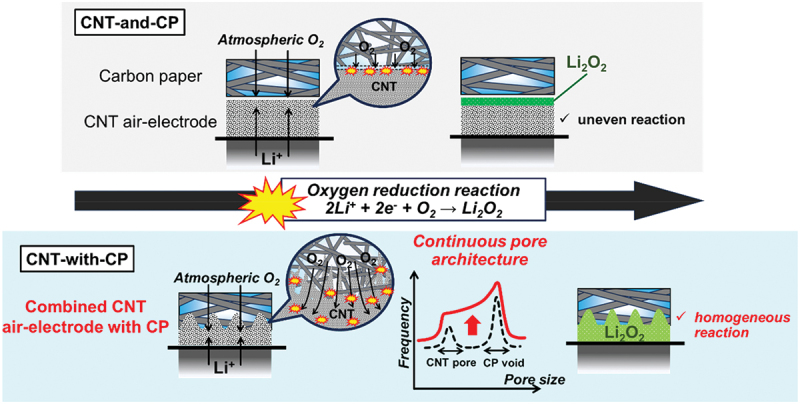
Schematic of ORR in CNT air-electrode.

Figure 5 shows the schematic of the ORR inside the CNT air-electrode. In CNT-and-CP configuration, ORR concentrates on the gas side surface of the CNT electrode, which clogs the CNT pores to terminate the cell discharge early. When the current rate is low or O_2_ gas concentration is high, the cell may still proceed ORR to generate discharge power, but this is not the ideal case of cathode reaction. In the case of CNT-with-CP configuration, the roughened gas side surface of the air-electrode, which is physically supported inside the CP layer and characterized by continuous pore architecture, keeps the ventilation pores open. This results in homogeneous ORR inside the air-electrode and enables discharge under air O_2_. However, the integrated CNT/CP selects the appropriate combination of the two layers. First, the CNT slurry needs to be properly prepared: poor CNT dispersion in the slurry medium results in unsuccessful preparation of a flat and uniform electrode layer, but a CNT dispersion that is too fine makes the CNT component slip out of the CP and fail to form a CNT layer on the CP. Water without any surfactants was selected as the CNT slurry medium, and the bloated CNT flocculates in the medium secured the fabrication of clean and flat CNT layer on CP. The CP specification is also responsible for realizing the ideal CNT/CP integration. Along with CP1 with high porosity (0.94), two other CPs, designated CP2 (TGP-H-030, Toray, Japan) and CP3 (TGP-H-060, Toray, Japan), which have a lower porosity (~0.80) and smaller void gaps (35 μm) than CP1 (130 μm), were filtrated with CNT slurries to form combinations denoted as CNT-with-CP2 and CNT-with-CP3, respectively. Despite the apparent integration, the CNT layers on CP2 and CP3 were easily detached from the CPs, indicating the unsuccessful bond between the CNT/CP layers. The XCT image of CNT-with-CP2 (Figure S10) revealed negligible penetration of the CNT component inside CP2, because the void width (35 μm) is one-fourth that of CP1 (130 μm). The pore distribution revealed by mercury intrusion (Figure S11) shows the two separated CNT pore and CP pore regions, like the case of CNT-and-CP1. The CNT/CP integration here fails to create a hierarchically continuous pore structure like that of CNT-with-CP1, and this structure therefore behaves as a single free-standing CNT layer like CNT-and-CP1 when used as an LAB cathode. As a result, CNT-with-CP2 and CNT-with-CP3 cells (Figure S12) exhibited lower capacities than that of the CNT-with-CP1 cells. The capacities in Li–air obtained with CNT-with-CP2 and CNT-with-CP3 were only 50–70% of those in Li – O_2_, and thus these cells cannot function as true LAB cells in atmospheric O_2_. The discharges of the CNT-with-CP2 and CNT-with-CP3 cells are better than that of CNT-and-CP1, which is because the CNT/CP pores are more closely matched by CP2 and CP3 (with 35 μm pores) than by CP1 (with 130 μm pores). In addition, the CNT-with-CP3 cells provide slightly higher capacity than that of CNT-with-CP2 cells because of the higher CP thickness and hence with the easier O_2_ access in CNT-with-CP3. However, the integrating CNT/CP layers does not develop a hierarchically continuous pore architecture in these cases and thus is not suitable as Li-air cathode. By using high porosity CP with wide void spacing, such as CP1, CNTs partially infiltrate the CP, forming hierarchical pores. This results in the creation of an air-electrode with high O_2_ transport efficiency.

### 3.3. Battery performance

**Figure 6.**
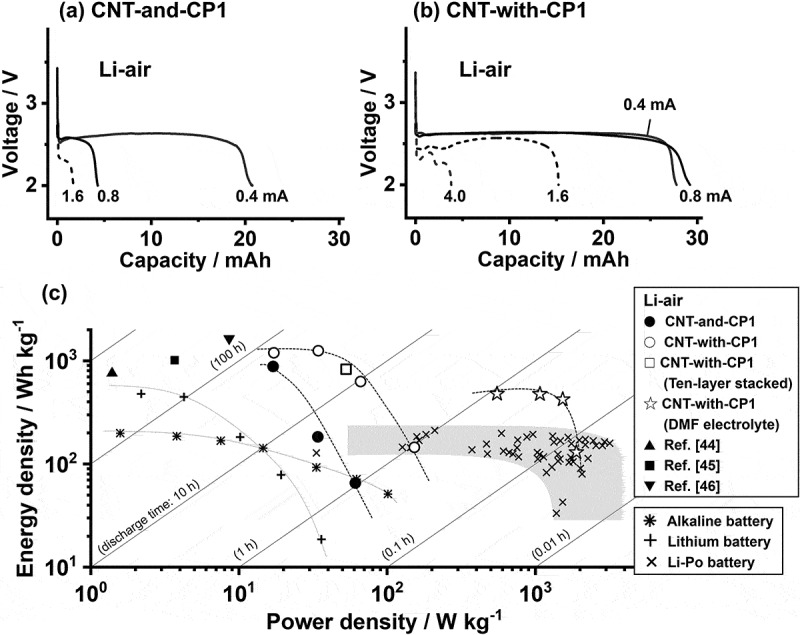
Discharge curves of (a) CNT-and-CP1 and (b) CNT-with-CP1 cathode cells under dry air (Li – air). The total mass of cell materials is 60.6 mg. (c) Battery cell energy density vs. power density (Ragone plot). The data points indicated by ● and ○ for Li – air cells are derived from the discharge curves in (a) and (b), and points marked as ☆ are from the discharge curves in Figure S13 using CNT-with-CP1 cathode with DMF-based electrolyte. The □ symbols represent the data for the 10-layer stacked lab cell in Figure 8. The ▲, ■, and ▼ symbols are Li-air cells from Li et al. [44], Fang et al. [45], and Yu et al. [46], respectively (Table S2). The data points for alkaline (*) and lithium (+) primary batteries are derived from the discharge curves in Figure S13. The data points for Li–Po (×) are from the literature [47] with 25 wt% deduction [48]. All plots are based on the core cell materials weight (total mass of lithium anode, cathode, separator, and electrolyte), excluding the pouch package and current collecting materials.

The successful LAB discharge under atmospheric O_2_ opens the possibility of realizing a true LAB working in atmospheric O_2_, which exists everywhere, unlike artificially concentrated O_2_ from gas cylinders or O_2_ enrichers that consume external power and add to the system mass [10]. To evaluate the discharge performance of the LAB cells with CNT air-electrodes, a rate-dependent discharge test was conducted in Li – air. Figure 6(a) shows the discharge curves of CNT-and-CP1 cells at currents of 0.4, 0.8, and 1.6 mA. Though the capacities are insignificant at relatively high currents of 0.8 or 1.6 mA, the low current of 0.4 mA yields a considerable cell capacity (20.8 mAh), suggesting that O_2_ inhalation is the rate-determining step. The cells with CNT-with-CP1 (Figure 6(b)) have significantly improved O_2_ inhalation, which increases the cell capacity at high currents, providing 15.2 mAh at 1.6 mA and 3.8 mAh even at 4.0 mA. The cell capacity is approximately 30 mAh at both 0.8 mA and 0.4 mA, which indicates that this capacity is the maximum for the CNT air-electrodes to host the Li_2_O_2_ discharge product [23]. The Li dissolution capacity of the Li anode used here (83 mAh for 16 mm diameter lithium foil with 200 μm thickness) surpasses the cell capacities in Figure 6. Because the anode resistance changes negligibly through Li dissolution under both pure O_2_ and dry-air conditions [24], the CNT cathode architecture exclusively governs the cell capacity here. These results suggest that the successful CNT-with-CP1 discharge under atmospheric O_2_ results from the enhanced current capability, which reduces the O_2_ adsorption barrier at the gas side of the air-electrode.

The discharge performance above was normalized with respect to the total mass of the cell materials, specifically 60.6 mg as the total mass of the Li foil (21.2 mg), separator (3.2 mg), electrolyte (24.0 mg), CNT air electrode (4.2 mg), and CP1 (8.0 mg), which are the core cell materials barest necessities for assembling an LAB device. Figure 6(c) shows the gravimetric energy density plotted against the power density (Ragone plot) to compare the discharge performances of various energy-storing devices. The plot data for lithium – polymer (Li – Po) batteries, which are commercial batteries rechargeable via LiB technology for small appliances, were obtained from the report by Stux et al. [47]. The data for an 11 g AAA alkaline battery and a 3.2 g CR2032 Li battery were derived by discharging these commercial batteries at several currents (Figure S13). To make a fair cross-technological comparison, the masses of current collectors and pouch envelopes, 3.2 g for the AAA alkaline battery, 1.7 g for the CR2032 Li battery, and 25 wt% for a Li – Po battery [48], were deducted from the battery masses to calculate the energy and power densities based on core cell materials weight. The graph shows that the LAB cells with CNT-and-CP1 (●) and CNT-with-CP1 (○) cathodes achieve an energy density as high as 1200 Wh kg^−1^ under dry air (Li – air), which is 5–10 times higher than that of Li – Po batteries (×, 100–200 Wh kg^−1^) and alkaline batteries (*, <200 Wh kg^−1^), and twice that of a Li battery (+, <500 Wh kg^−1^) or higher. Some studies report LABs with energy densities of ~1000 Wh kg^−1^ [2–5], but they operate only under a pure O_2_ atmosphere (Li – O_2_), so their practical usefulness is questionable. The Ragone plot here suggests that such a high energy density (~1000 Wh kg^−1^) is entirely possible even under atmospheric air. It should be noted that, because the mass of the LAB cells (60.6 mg) is quite different from that of commercial battery devices, the plot data indicate the best estimates of the energy and power achievable by ideally scaling up the cell configurations in Figure 1 to an Ah-class device level. However, the validity of this projection is supported by the data point □ (830 Wh kg^−1^), which is for a 10-layer stacked cell discussed later (4.7 g based on core cell materials weight) and is near the data curve for CNT-with-CP1 cells (○).

As with battery energy, battery power is also a critical metric for determining the practicality of this battery technology. Although both the CNT-and-CP1 (●) and CNT-with-CP1 (○) cathodes provide energy in the 1000 Wh kg^−1^ class, the CNT-with-CP1 curve extends approximately three times as far along the power density axis as CNT-and-CP1. This is because the continuous pore architecture in CNT-with-CP1 effectively adsorbs external O_2_ gas, enabling a high-current discharge in Li – air. Though the O_2_ in atmospheric air is one-fifth of pure O_2_, it is not actually difficult to discharge LABs under air at low discharge power conditions [44–46,49]. It has been recently reported that polymer-based electrolyte that protects lithium anode from air moisture [44,45], or multi-bifunctional catalyst cathode not only for Li-O_2_ but also for Li-CO_2_ and Li-H_2_O electrochemistry [46], enables ambient air operation of LAB cell devices for a few hundred hours, delivering the 1000 Wh kg^−1^ class energy (▲, ◼, and ▼, the discharge data are tabulated in Table S2). However, the applied current less than 10 mA per few grams of cell device limited the power density to no more than 10 W kg^−1^, restricting its potential application only to low-power appliances such as LEDs. Batteries for general use must have much more power. In that context, CNT-with-CP1 with its hierarchically continuous pore structure plays a crucial role in enhancing the LAB power. With that said, the CNT-with-CP1 cells still have the power levels of alkaline or Li batteries (<100 W kg^−1^), which are insufficient for powering high-power appliances such as EVs or drones that are currently powered by LiB technology. Further enhancing the power requires additional attempts in electrolytes along with cathode engineering. We have recently found a high discharge rate of LAB cells with the CNT-with-CP1 cathode by replacing the TEG-TFSI electrolyte with low-viscosity amide-based electrolytes [24]. For example, *N,N*-dimethylformamide (DMF) dissolving 1.0 M of LiNO_3_ salt (DMF-NO_3_) enables stable discharges even at currents of >40 mA by cells with a CNT-with-CP1 cathode and a 50 μm thick Li foil anode (Figure S13), with a total core cell materials mass of 44.7 mg. This corresponds to power densities greater than 1000 W kg^−1^, which yield an energy density of 480 Wh kg^−1^ (☆). The high volatility of the DMF solvent at the cell’s air inlet inevitably shortens the discharge time, resulting in a lower battery energy than with the TEG-TFSI electrolyte [22]. However, low-viscosity amide-based electrolytes accelerate the transport of dissolved O_2_ and Li^+^ inside the air electrode, synergistically enhancing the rate along with the hierarchically porous electrode. Because of this, LABs have potential applications as power sources for mobilities, providing 5–10 times higher battery energy than LiBs at a power similar to that of LiB technology. The battery energy at this high power would be further improved by cell engineering that reduces the electrolyte dry-out, thus securing air O_2_ inhalation. This is the birth of new battery technology that is much lighter but has much higher energy and power than any previous battery technologies.

**Figure 7.**
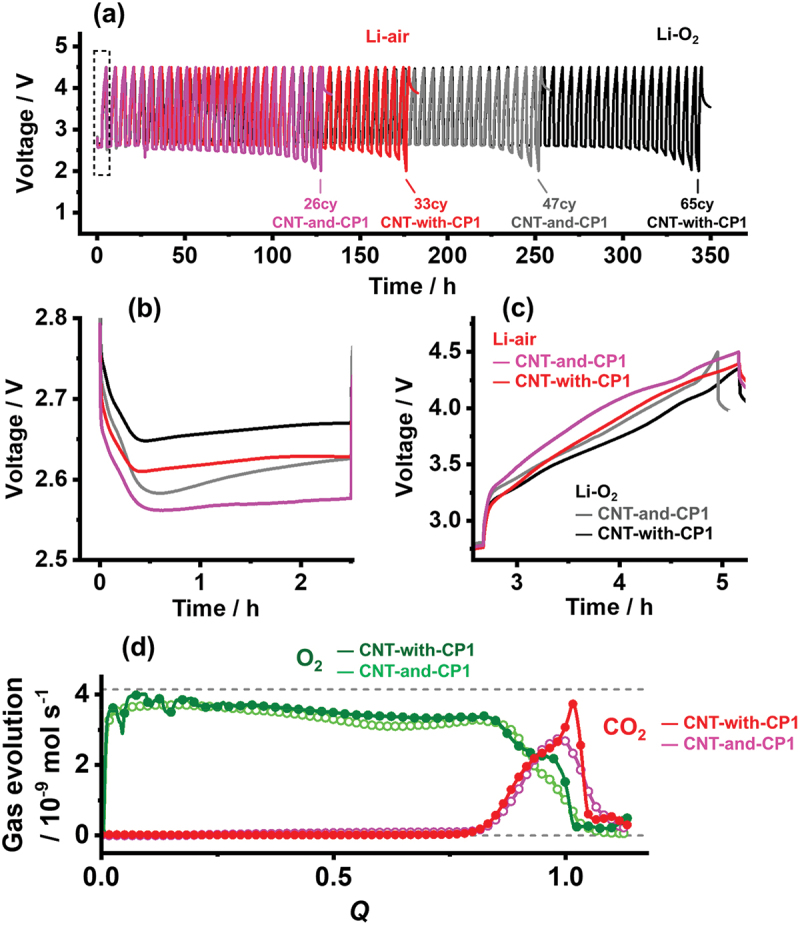
(a) Discharge and charge curves of CNT-and-CP1 (gray and pink) and CNT-with-CP1 (red and black) cells in Li – O_2_ (gray and black) and Li – air (pink and red) for a cycling condition of 0.4 mA × 2.5 h. The numbers in the graph indicate the cycle number, which is the number of discharges that provided the curtailed cycle capacity of 1.0 mAh. (b) Enlarged discharge and (c) charge curves for the first cycle runs for the dashed rectangular region in (a). (d) O_2_ and CO_2_ evolution curves during charging at 0.8 mA recorded after a discharge of 0.8 mA × 5 h. *Q* represents the charging capacity normalized with respect to a discharge capacity of 4 mAh. The dashed lines represent the ideal O_2_ gas evolution based on a 2e^−^/O_2_ reaction at currents of 0.8 mA (up) and 0 mA (down). The accumulated O_2_ and CO_2_ amounts at *Q* = 1.1 were 61.6 and 8.8 μmol, respectively, for CNT-with-CP1 and 58.2 and 8.5 μmol, respectively, for CNT-and-CP1. The discharge and charge curves for the gas analysis are shown in Figure S16.

The cycle performance was also evaluated to assess the practicality of these cells as rechargeable systems. Figure 7(a) shows the repeated discharge – charge cycle profiles of the CNT-and-CP1 and CNT-with-CP1 cathode cells under dry air (Li – air) and pure O_2_ (Li – O_2_). To make a comprehensive comparison between the air-electrodes in Li – air and Li – O_2_, the repeated-discharge condition was set to 0.4 mA × 2.5 h (corresponding to approximately 40 Wh kg^−1^), much lower than their discharge capability of ~1000 Wh kg^−1^. At this cycle capacity (1.0 mAh), Li/Li symmetric cell bears more than 100 cycles with no obvious increase in overpotential [50]. Therefore, the limited cycle life below 100 cycles in Figure 7(a) can be attributed to the failure of cathode-related battery reactions. The profiles reveal that the cycle life of cells in Li – air is half of that in Li – O_2_. This is ascribed to the limited O_2_ and Li^+^ transport in the TEG-TFSI electrolytes compared with that in the low-viscosity amide-based electrolytes, which enables similar cycle lives in both Li – air and Li – O_2_ [24]. However, the profiles reveal that the cycle life of the CNT-with-CP1 cells is 1.4 times longer regardless of the O_2_ gas concentration. This results from the more homogeneous ORR behavior inside the CNT-with-CP1 air electrode during the whole cycle life, which effectively suppresses the electrode degradation arising from the localized reaction. The improved cycle life for CNT-with-CP1 can also be inferred from the very first cycle run (Figure 7(b,c)), because the cells exhibit lower overpotentials both in discharging and charging. The energy efficiencies of the CNT-with-CP1 cells (72% in Li – O_2_ and 68% in Li – air) are higher than those of the CNT-and-CP1 cells (69% in Li – O_2_ and 65% in Li – air), suggesting a lower rate of material degradation in CNT-with-CP1 cells. However, the energy efficiencies are much lower than those of current LiB technology (>80–90% [51]), which is due to the high overpotentials especially during charging. XRD spectra of CNT-with-CP1 after discharge (Figure S14) suggest Li_2_O_2_ as a main discharge product under both Li-O_2_ and Li-air conditions. The spectra imply slightly lower crystallinity of Li_2_O_2_ in the Li-air condition than in the Li-O_2_ condition. The cell weight change during discharge-charge (Figure S15) exemplifies an ideal 2e^−^/O_2_ reaction during cycle operation, except for excessive gas evolution near the end of charge. These facts indicate that the ideal LAB cathode reaction (2Li^+^ + O_2_ +2e^−^ ↔ Li_2_O_2_) proceeds during discharge at least, but that the cells undergo unexpected side reactions during charge. Figure 7(d) shows the gas evolution profiles during the first charge of CNT-with-CP1 and CNT-and-CP1 cathode cells (the voltage profile for the gas analysis is shown in Figure S16). Although the O_2_ evolution efficiency for CNT-with-CP1 (81% of the ideal O_2_ evolution) is improved from that of CNT-and-CP1 (78%), the profiles reveal considerable CO_2_ evolution in both cathode cells that explains the excessive outgassing near the full charge. This indicates significant oxidative decomposition of the electrolyte and the carbon electrodes even when CNT-with-CP1 is used, limiting the number of cycles to less than 100. This is unfortunately much lower than that of the current LiB technology, which operates for over 10^3^ cycles. As a rechargeable battery device, the LAB faces challenges not only with carbon-based cathodes but also with the lithium and electrolyte stabilities. Therefore, it is currently difficult to assemble LAB cells with a sufficient cycle life, but cathodes with hierarchically continuous pore structure provide the best platform once these challenges are resolved.

**Figure 8.**
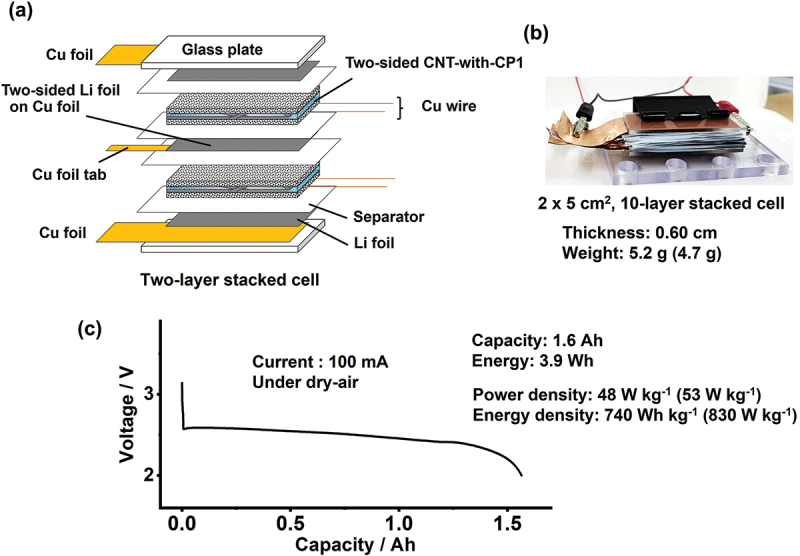
(a) Schematic of a two-layer stacked Li – air cell using double-sided CNT-with-CP1 cathodes. A cross-sectional SEM image of the double-sided CNT-with-CP1 is shown in Figure S17. (b) Photograph of a 10-layer stacked Li – air cell. The exact stacking configuration is shown in Figure S18. (c) Discharge curve of the 10-layer stacked Li – air cell at a current of 100 mA. The numbers in parentheses indicate the core cell materials weight excluding the weight of the Cu current collectors, and the energy and power densities based on the core cell materials weight.

Though the cycle life is unsatisfactory, the high energy density of the LAB with its high-power output gives it a technological edge over any existing battery technologies. This should be verified by scaling up small cells (Figure 1) to test whether they can form Ah-class battery devices. To this end, a multiple-electrode stacked cell was fabricated. Figure 8(a) shows the schematic of a two-layer stacked cell, in which a two-sided 75 μm thick Li foil on a 6 μm thick Cu foil (obtained from Honjo Metal) is sandwiched by two separators and two double-sided CNT-with-CP1 cathodes. This stack was bookended by a spring clamp between two glass plates, each of which was taped to a Cu foil and a Li foil. Each double-sided CNT-with-CP1 was prepared by filtering a CNT slurry again on the CP1 surface of one-sided CNT-with-CP1. Because the CNT/CP layers of CNT-with-CP1 are firmly combined, the second CNT slurry can be filtrated on the other CP1 surface without delaminating the first CNT layer, yielding CNT layers on both CP1 surfaces. This double-sided cathode cannot be achieved with CP2 or CP3, which have low porosity. To collect current from the double-sided air-electrode, two Cu wires with a diameter of 50 μm were sewn on CP1, which had dimensions of 2 × 5 cm^2^, before the CNT slurry filtration. Figure S17 shows the cross-sectional SEM image of the double-sided CNT-with-CP1, demonstrating the integrated CNT/CP/CNT layers with Cu wires embedded inside CP1. Here, we use Cu as a positive current collecting material for a discharge-only LAB device, but if repeated discharge via charging is anticipated, an alternative material such as aluminum may be preferable in order to prevent the accidental dissolution of Cu.

To derive Ah-class capacity, a 10-layer stacked cell with rectangular electrode dimensions of 2 × 5 cm^2^ was fabricated, as shown in Figure 8(b). To achieve ionic conductivity, the electrodes and separators were wetted with 12 μL cm^−2^ of electrolyte per electrode area. The total active electrode area was 200 cm^2^ and the stack mass was 5.2 g (4.7 g excluding Cu current collecting materials) with a thickness of 0.60 cm under 118 kPa pressure (the exact stacked configuration is illustrated in Figure S18, and the mass of each cell component is provided in Table S3). Figure 8(c) is the discharge profile of the 10-layer stacked cell at a current of 100 mA (current per electrode unit area of 0.5 mA cm^−2^), delivering a capacity of 1.6 Ah (energy of 3.9 Wh) under dry air. This corresponds to a battery cell with an energy of 740 Wh kg^−1^ with a power output of 48 W kg^−1^, successfully demonstrating a small battery device with high energy density based on LAB technology working in atmospheric O_2_. Based on the mass of core cell materials without Cu current collecting materials (4.7 g), it was 830 Wh kg^−1^ energy at 53 W kg^−1^ power (◻ in Figure 6(c)), closely located near the energy/power trend for CNT-with-CP1 cathode cells (○ in Figure 6(c)). The battery specifications exceed the reported powers of LABs under air [40,44–46] with securing the ~1000 Wh kg^−1^ class energy. Slight decrease in energy density from the trend of the tested battery cells with CNT-with-CP1 resulted from the misalignment of some electrodes and the shortage of lithium source for discharge. However, the Ah-class capacity delivered by the 10-layer stacked cell demonstrates that the stacked LAB cells can be scaled to a battery device. Precise cell-assembling technology with further development of air-electrodes based on hierarchically porous architecture will realize LAB devices for practical application.

## 4. Conclusion

Despite the high energy density of LABs, it is difficult to actually discharge them in air. To find the discharge obstacles, this study investigated the ORR inside an air-electrode consisting of a highly porous CNT sheet. The cross-sectional SEM observation and EDS elemental analysis revealed that the ORR gradually penetrates the gas side and progresses to the opposite separator side of the sheet air-electrode. The reaction becomes more inhomogeneous in a low-O_2_ environment such as atmospheric air, which blocks continuous O_2_ permeation, thus prematurely stopping the cell discharge. An approach to avoid the reaction concentration at the gas side surface is developing a hierarchically continuous pore architecture in the air-electrode that is configurable by combining the CNT air electrode with a CP GDL, which was demonstrated by the CNT-with-CP1 cathode prepared by filtering a CNT slurry on CP1. XCT observation and mercury intrusion porosimetry revealed the creation of a continuous pore structure on the gas side surface of the CNT air-electrode layer, smoothly connecting nanopores for ORR and micrometer-sized voids for O_2_ inhalation. This continuous pore architecture enables LAB cells with the integrated cathode to discharge at a high rate under atmospheric O_2_, which is scalable for configuring an Ah-class battery device that can operate under dry-air. Though the cycling capability is limited, the integrated cathode prolongs the cycle life of LAB cells. All experiments in this study were conducted in dry conditions (pure O_2_ or dry-air) to eliminate the chance of hygroscopic degradation of the cells. For the development of true LAB, however, further investigation is needed to understand the effect of air quality (temperature and barometric pressure) and gas impurities (H_2_O and CO_2_) that are known significantly affect the battery reaction on factors including battery safety [9,11]. These investigations have solely relied on feasible ORR systems driven by atmospheric O_2_, necessitating LAB cells that operate at ambient O_2_ concentrations rather than in pure O_2_. An air-electrode with continuous pore architecture contributes to this practical development, providing the best platform for promoting an atmospheric ORR.

## Supplementary Material

Supplemental Material

## Data Availability

The data that support the findings of this study are available from the corresponding author upon reasonable request.
