# Supplementary material for: An air-electrode with hierarchically continuous pore architecture: a step toward “true” lithium–air batteries working with atmospheric oxygen
Source: Sci Technol Adv Mater. 2026 May 8;27(1):2658329. doi: 10.1080/14686996.2026.2658329 (PMC13162549; doi:10.1080/14686996.2026.2658329)
Supplement: Supplemental Material [file TSTA_A_2658329_SM5789.docx]

**Supplemental materials**

**An air-electrode with hierarchically continuous pore architecture: a step toward “true” lithium–air batteries working with atmospheric oxygen**

Akihiro Nomura*^a,^* * and Kimihiko Ito*^a^*

*^a^* Research Center for Energy and Environmental Materials, National Institute for Materials Science, 1-1 Namiki, Tsukuba, Ibaraki 305-0044, Japan

*Correspondence and requests for materials should be addressed to NOMURA.Akihiro@nims.go.jp

**Table S1.** Characteristics of carbon papers (CPs) used in this study.

|  | CP1  (E704, Kureha) | CP2  (TGP-H-030, Toray) | CP3  (TGP-H-060, Toray) |
| --- | --- | --- | --- |
| Sheet resistivity *^a^*  / Ω sq. | 3.3 × 10^0^ | 6.4 × 10^-1^ | 2.8 × 10^-1^ |
| Carbon fiber diameter *^b^* / μm | 14 - 20 | 7 – 8 | 7 - 8 |
| Sheet thickness *^c^* / μm | 300 | 110 | 190 |
| Sheet weight  / mg cm^-2^ | 4.0 | 4.2 | 8.2 |
| Sheet density, *ρ* *^d^*  / g cm^-3^ | 0.13 | 0.38 | 0.43 |
| Porosity *^e^* | 0.94 | 0.82 | 0.79 |
| Most frequent pore void width *^f^* / μm | 130 | 35 | 35 |

*^a^*Measured using a sheet resistivity meter. *^b^*Determined by SEM observation (Figure S1). *^c^*Catalog values. *^d^*Derived by dividing the sheet mass by the sheet thickness. *^e^*Calculated as 1− *ρ*/*ρ*_carbon_, where *ρ* is the sheet density and *ρ*_carbon_ is the real density of graphite carbon (2.1 g cm^−3^). *^f^*Determined by mercury porosimetry (Figure S2).


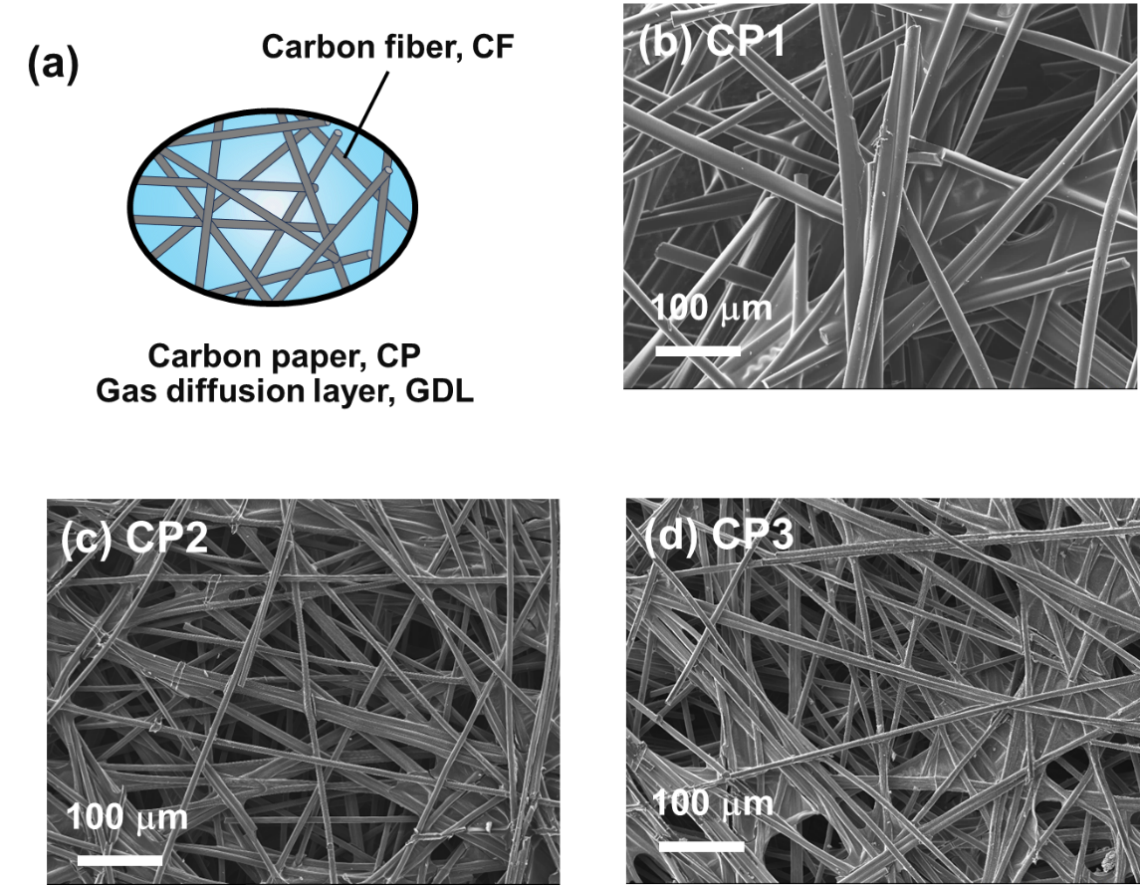


**Figure S1.** (a) Illustration of carbon paper (CP) composed of graphitic carbon fibers (CFs) piled in a sheet. Scanning electron microscopy (SEM) images of (b) CP1, (c) CP2, and (d) CP3.


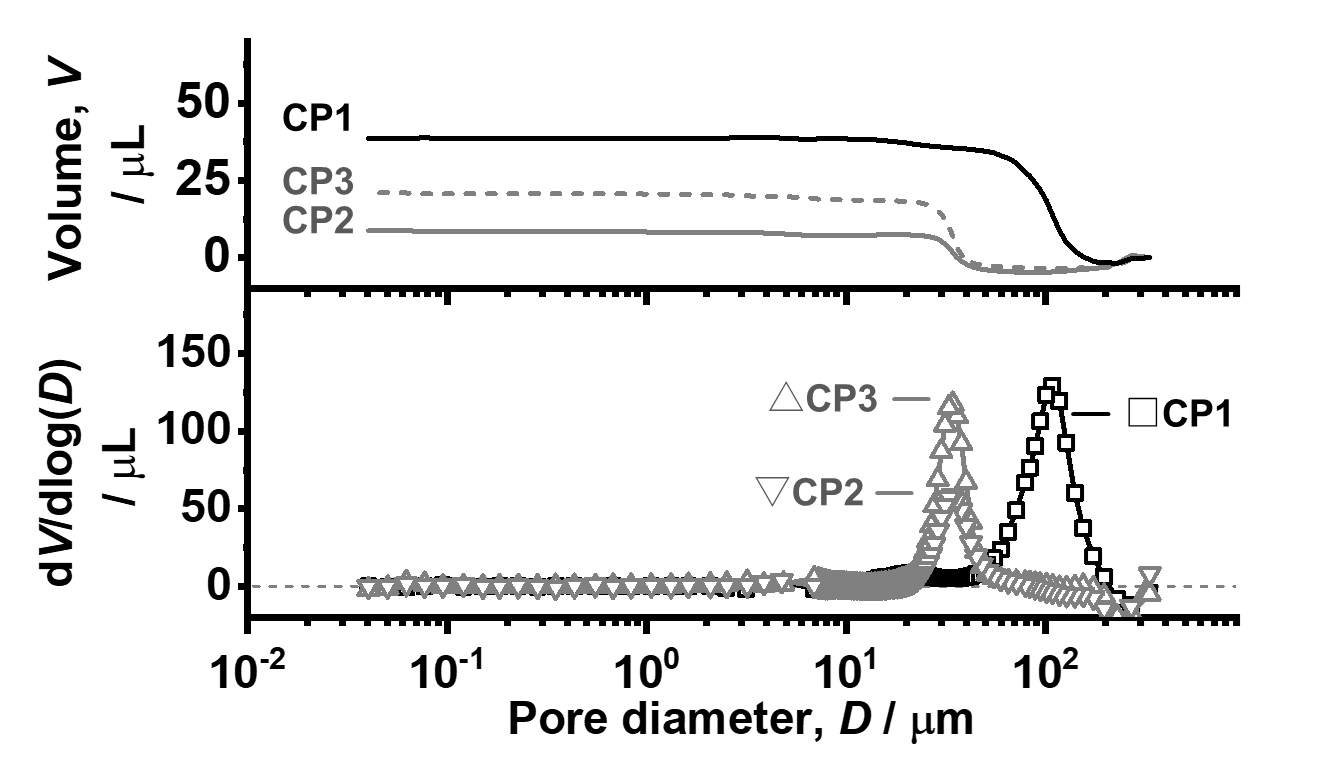


**Figure S2.** Pore size distributions (down) and cumulative pore volume (up) of CP1, CP2, and CP3.


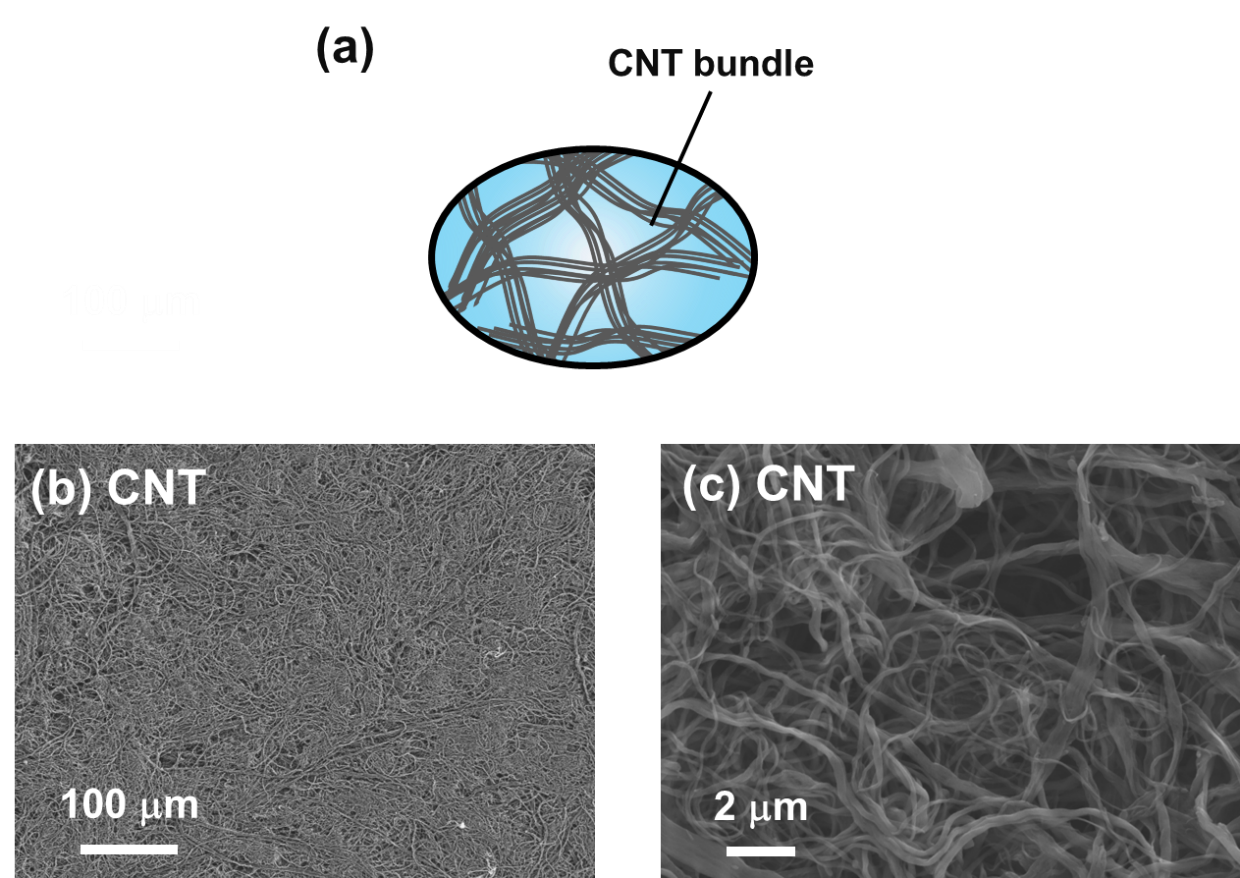


**Figure S3.** (a) Illustration of a carbon nanotube (CNT) sheet composed of CNT bundles aggregated in a non-woven textile. (b) SEM images of a single CNT sheet and (c) its magnification.


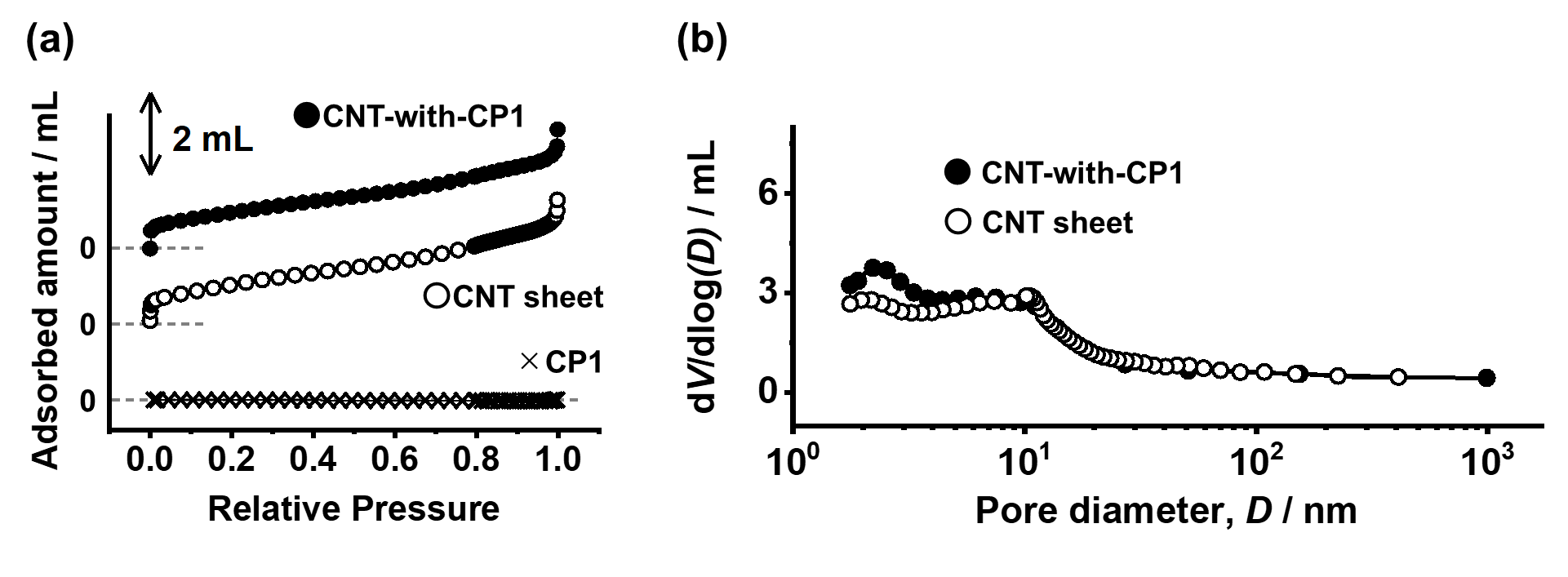


**Figure S4.** (a) N_2_ adsorption isotherms of CP1 (×), a single CNT sheet (○), and CNT-with-CP1 (●). The measurements were conducted for samples in a *φ*16 mm dimension (area of 2.0 cm^2^). The CNT loading for a single CNT sheet and CNT-with-CP1 was 4.2 mg (2.1 mg cm^−2^). (b) Pore size distributions of a single CNT sheet (○) and CNT-with-CP1 (●) obtained with the Barrett–Joyner–Halenda (BJH) model from the N_2_ adsorption isotherms in (a).


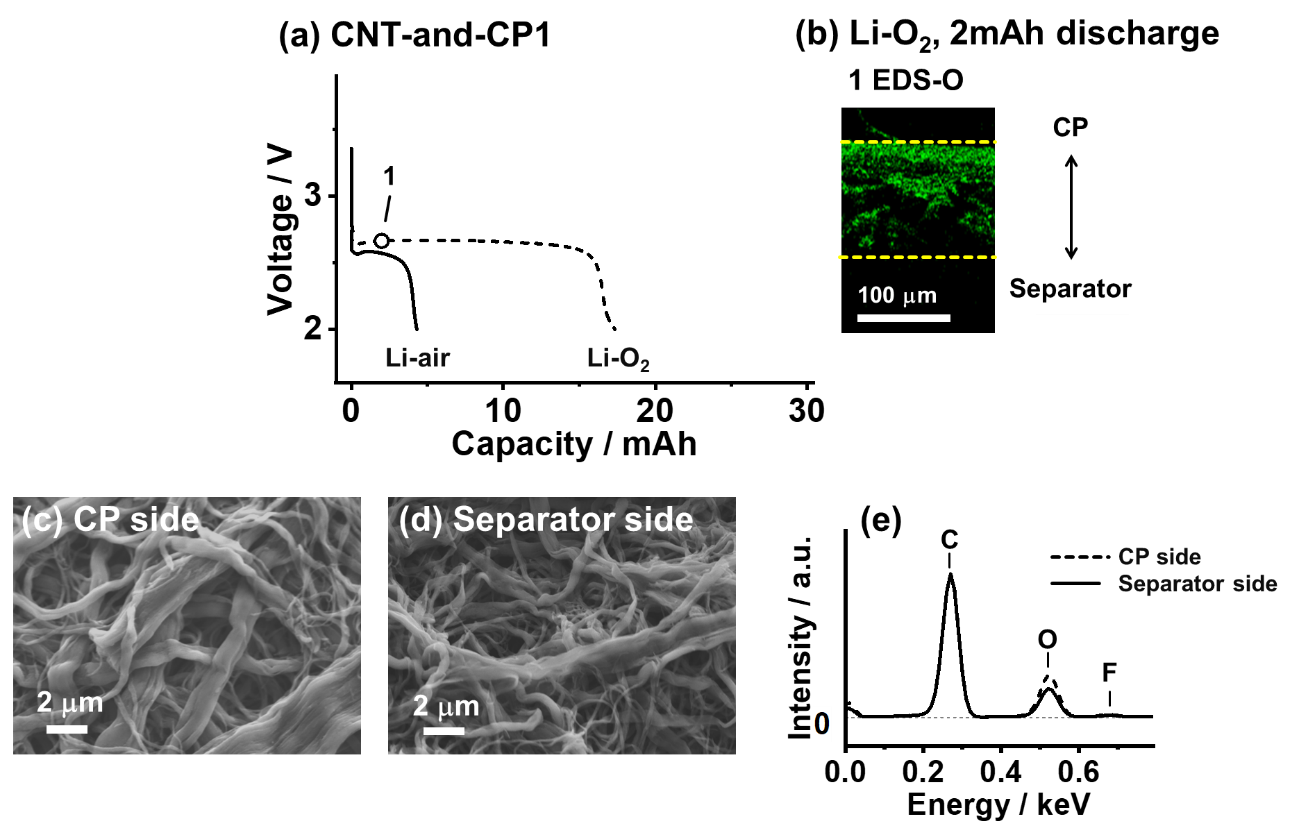


**Figure S5.** (a) Discharge curves of CNT-and-CP1 cathode cells (duplicate of Figure 2(a)). (b) Energy dispersive spectroscopy (EDS) elemental O mappings of CNT layer cross sections at point 1 in Li–O_2_ for a 2 mAh discharge (duplicate of Figure 2(b)). SEM images of the CNT surface of the (c) CP gas side and (d) separator side at point 1 in (a). (e) EDS spectra obtained from the SEM images in (c) for the CP side (dashed line) and (d) for the separator side (solid line). The peak intensities are normalized with respect to the C peak at 0.27 keV.


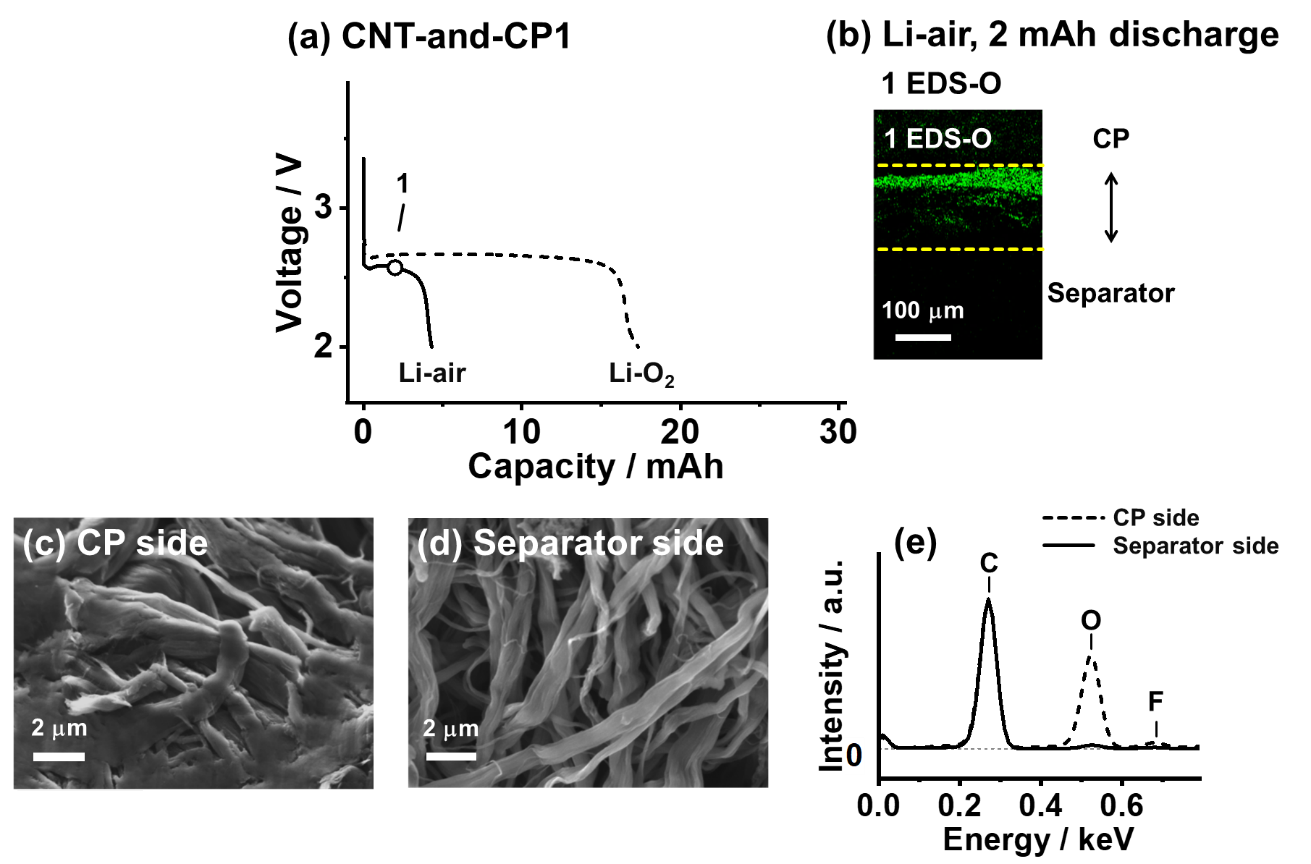


**Figure S6.** (a) Discharge curves of CNT-and-CP1 cathode cells (duplicate of Figure 2(a)). (b) EDS elemental O mappings of CNT layer cross sections at point 1 in Li–air for a 2 mAh discharge (duplicate of Figure 2(b)). SEM images of the CNT surface of the (c) CP gas side and (d) separator side at point 1 in (a). (e) EDS spectra obtained from the SEM images in (c) for the CP side (dashed line) and (d) for the separator side (solid line). The peak intensities are normalized with respect to the C peak at 0.27 keV.


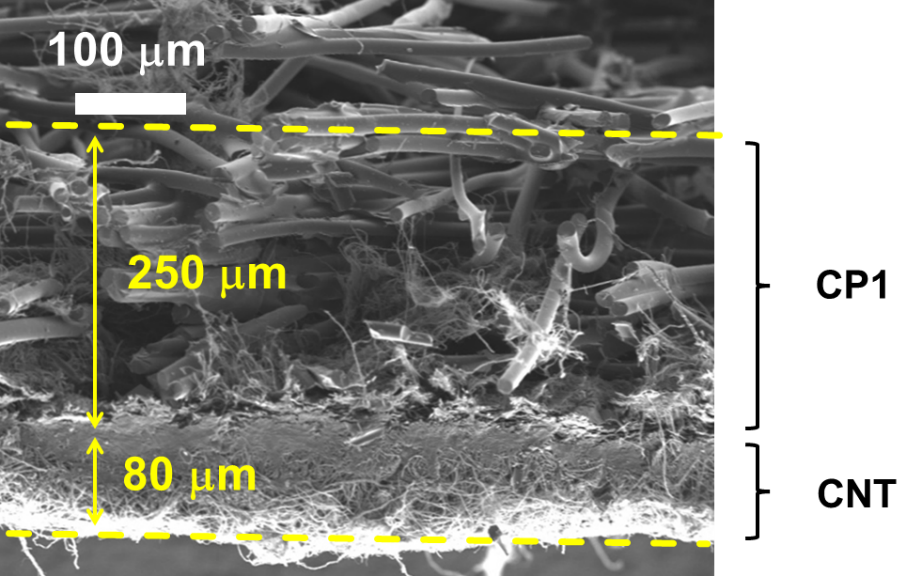


**Figure S7.** SEM image of the CNT-with-CP1 cross section. The dashed yellow lines indicate the bottom and top surfaces of the cross section.


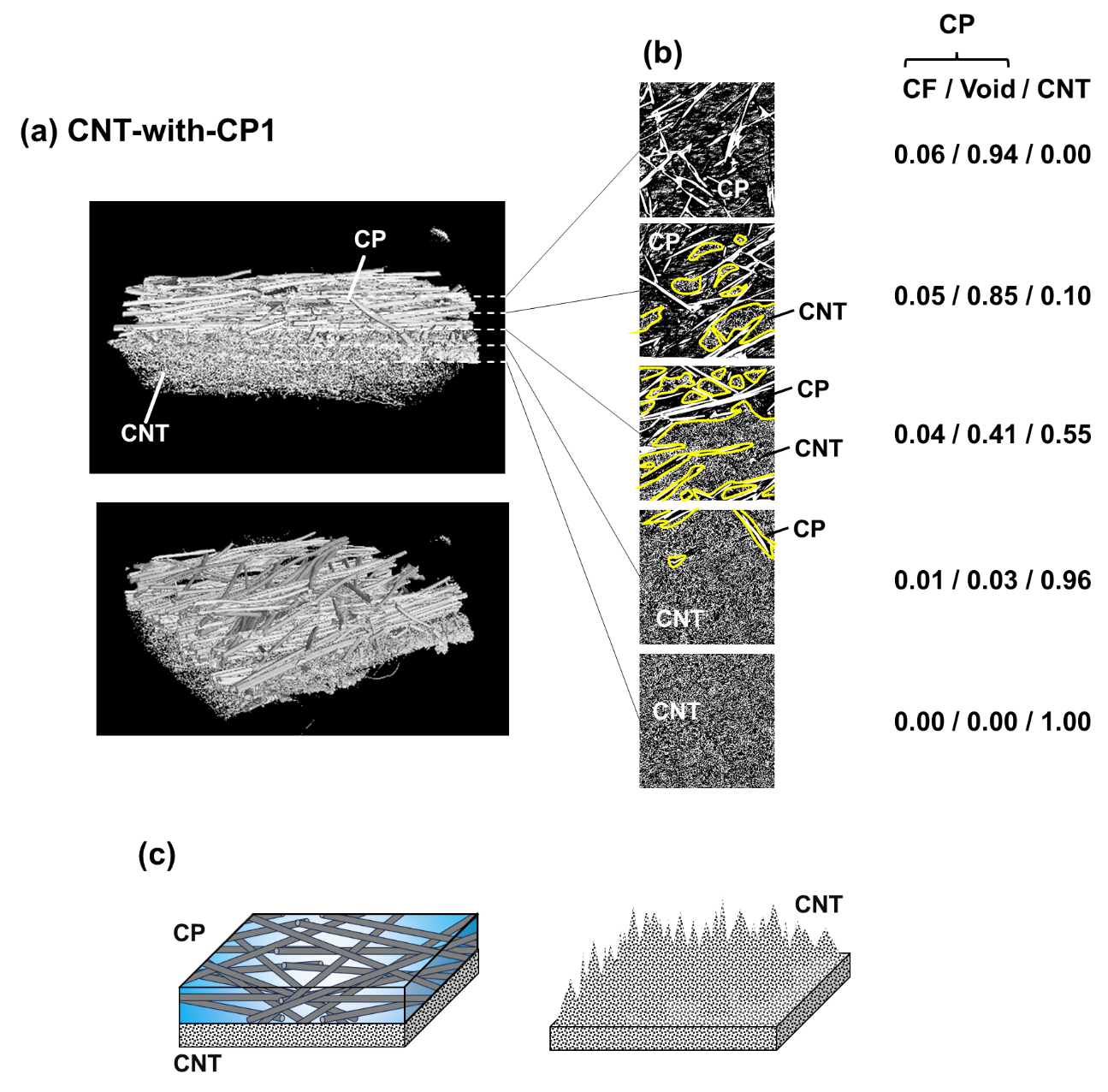


**Figure S8.** (a) 3D X-ray computed tomography (XCT) images of CNT-with-CP1. (b) 2D XCT images of the CNT-with-CP1 cross section parallel to the sheet face. The yellow lines in the images show the interface between the CNT and CP components. The numbers on the right indicate the volume fractions of the CF, CP void, and CNT components of each cross-sectional image. The CF/CP void ratio is adjusted to the porosity of CP1 (0.94). (c) Illustrations of CNT-with-CP1 structure. The CNT component infiltrates the CP1 layer, as shown on the right.


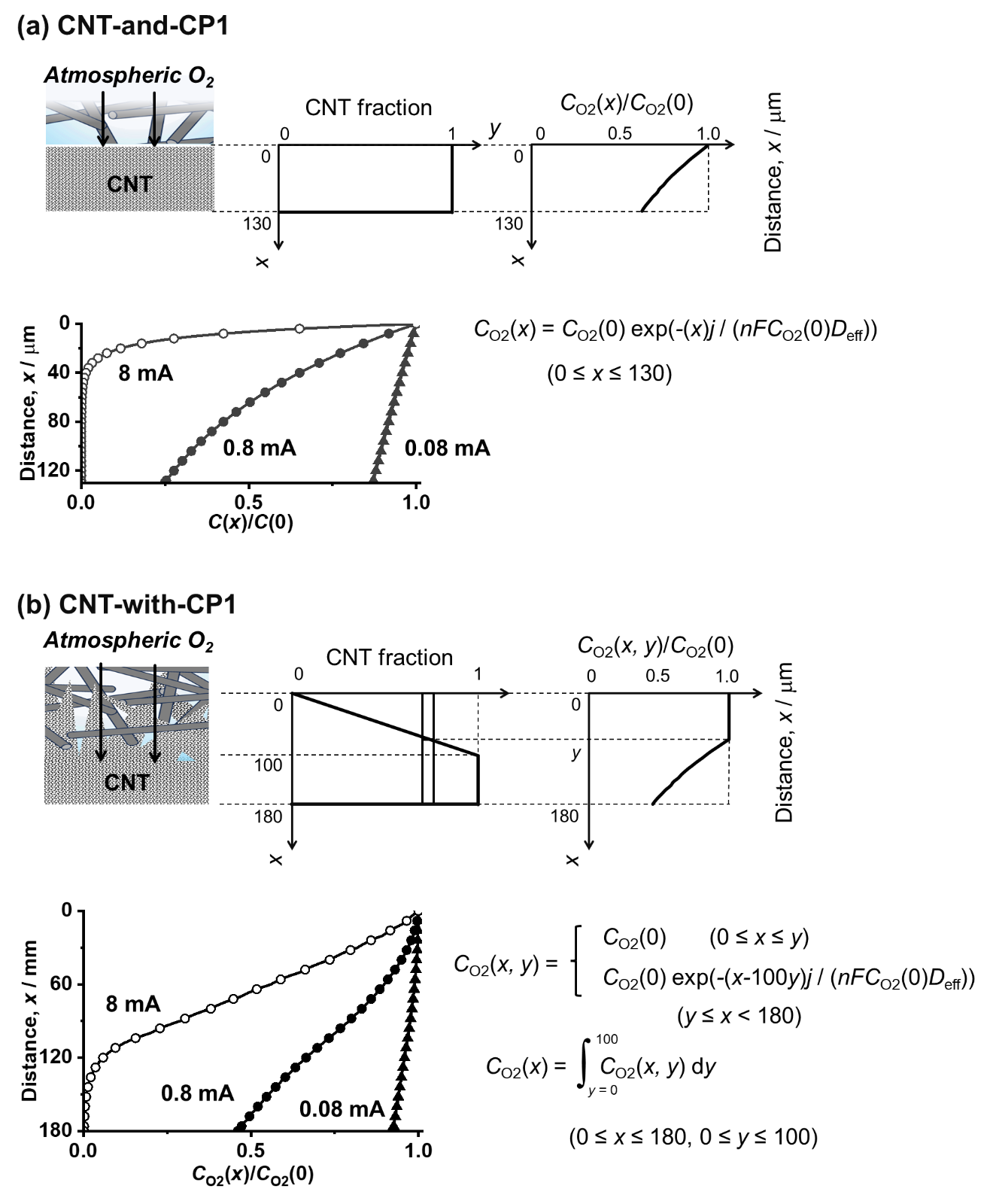


**Figure S9.** Conceptual scheme for the O_2_ concentration profile with the CNT air electrode thickness filled with electrolyte. (a) CNT-and-CP1, representing a non-continuous pore cathode. (b) CNT-with-CP1, representing a continuous pore cathode.


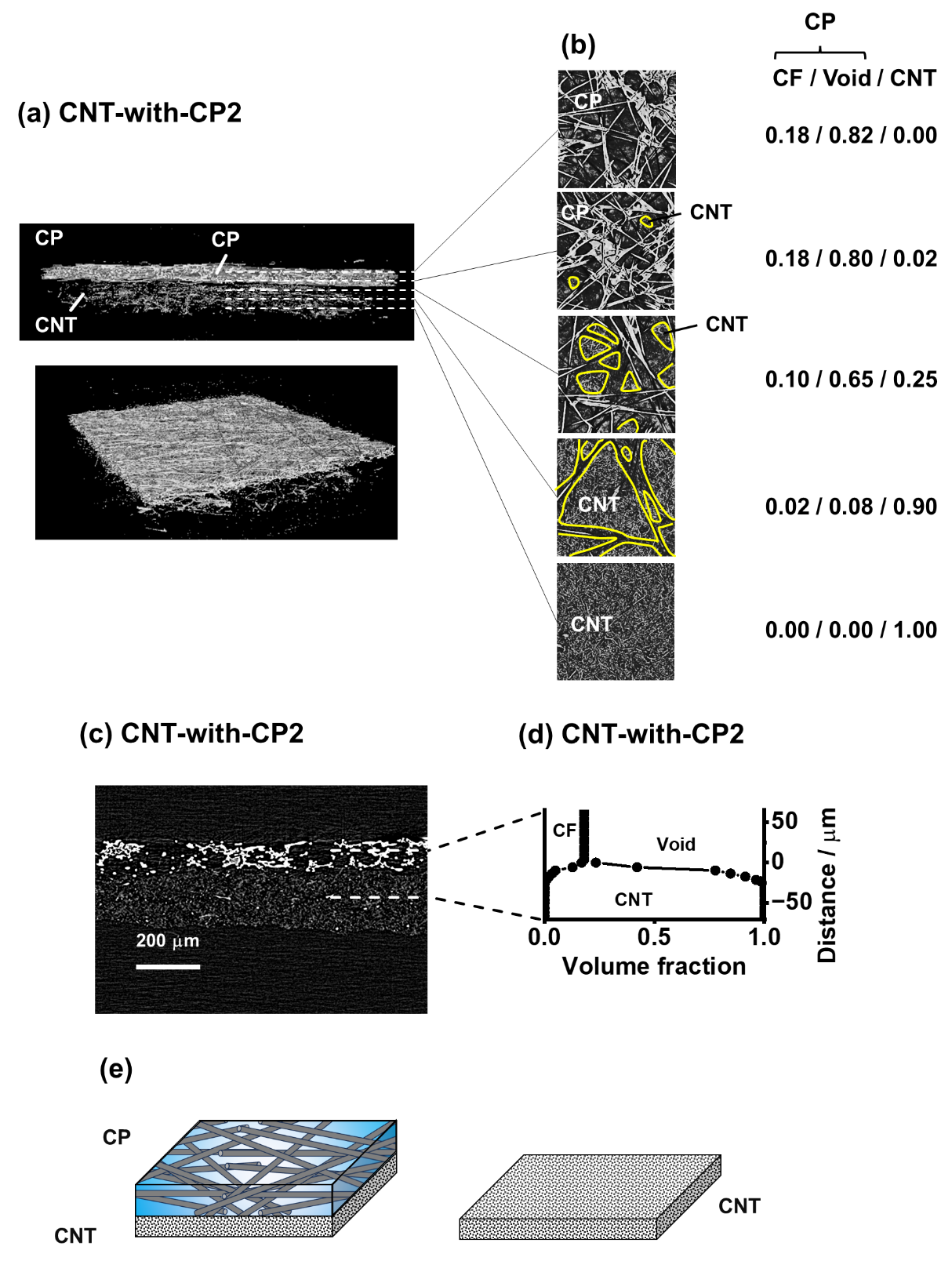


**Figure S10.** (a) 3D XCT images of CNT-with-CP2. (b) 2D XCT images of the CNT-with-CP2 cross section parallel to the sheet face. The yellow lines in the images show the interface between the CNT and CP components. The numbers on the right indicate the volume fractions of the CF, CP void, and CNT components of each cross-sectional image. The CF/CP void ratio is adjusted to the porosity of CP2 (0.82). (c) XCT cross-sectional image of the CNT-with-CP2 cathode. (d) Volume fractions of the CNT, CF, and void components near the interface of the CNT and CP2 layers. (e) Illustrations of CNT-with-CP2 structure. The CNT component does not significantly infiltrate the CP2 layer, forming a flat and smooth surface like a single CNT sheet, as shown on the right.


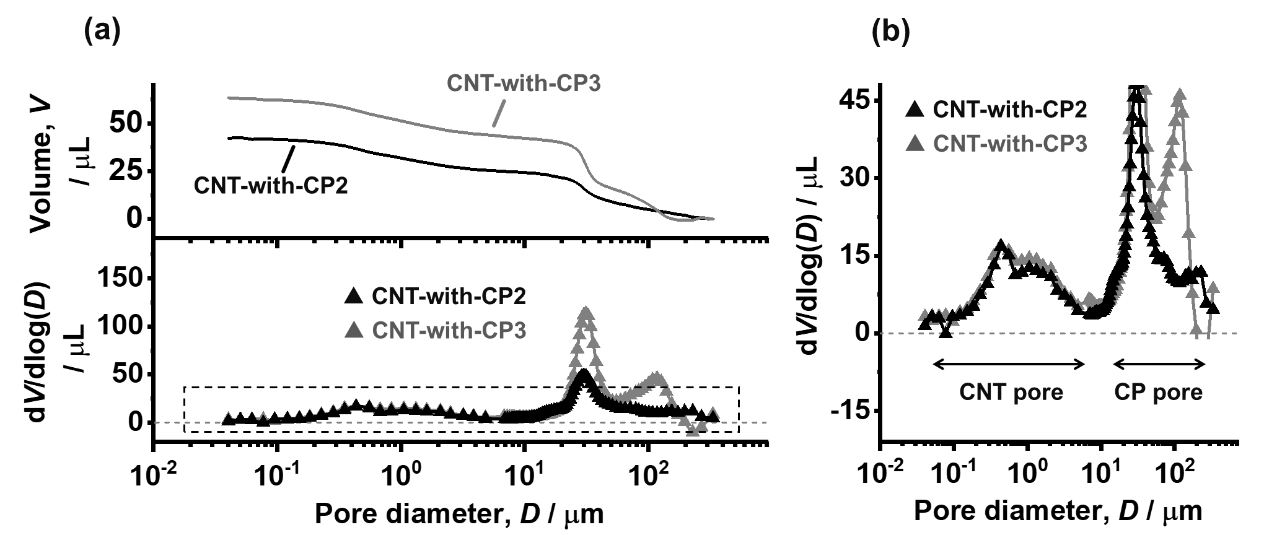


**Figure S11.** (a) Pore size distribution (down) and cumulative pore volume (up) of CNT-with-CP2 and CNT-with-CP3 cathodes. (b) Enlarged pore size distribution of the dashed rectangular region in (a).


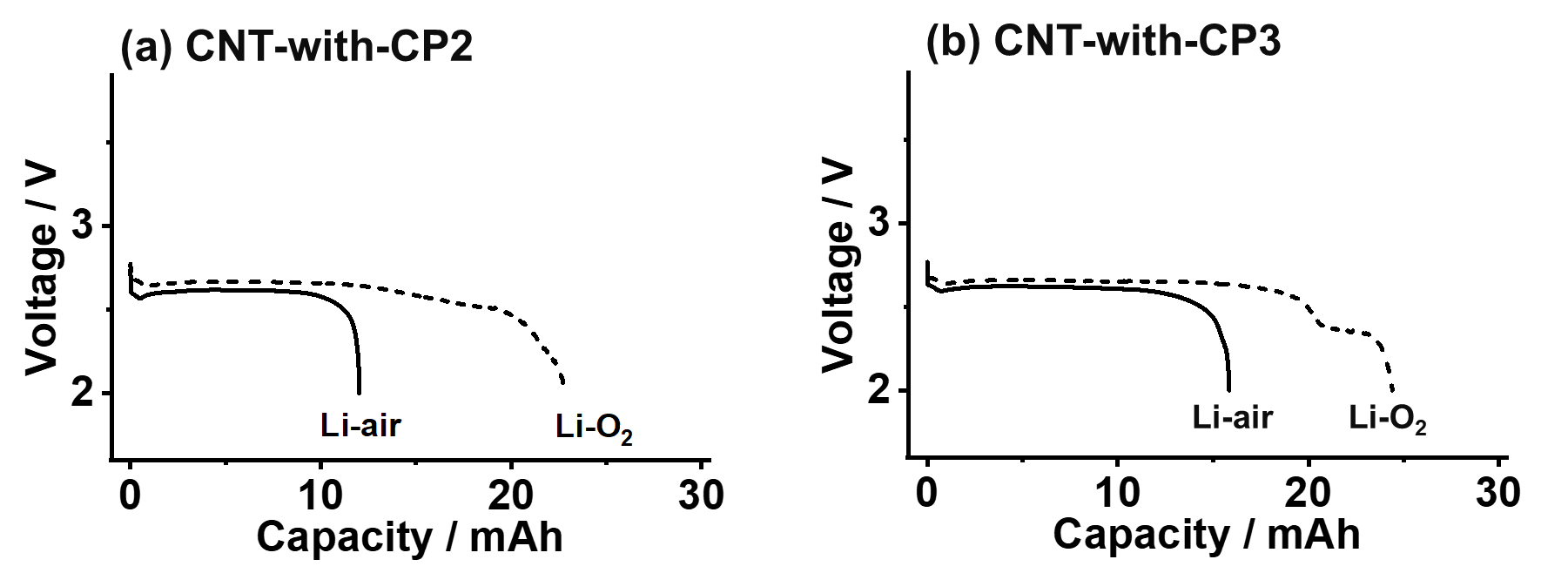


**Figure S12.** Discharge curves of (a) CNT-with-CP2 and (b) CNT-with-CP3 cathode cells under pure O_2_ (Li–O_2_) and dry air (Li–air) at a current of 0.8 mA.


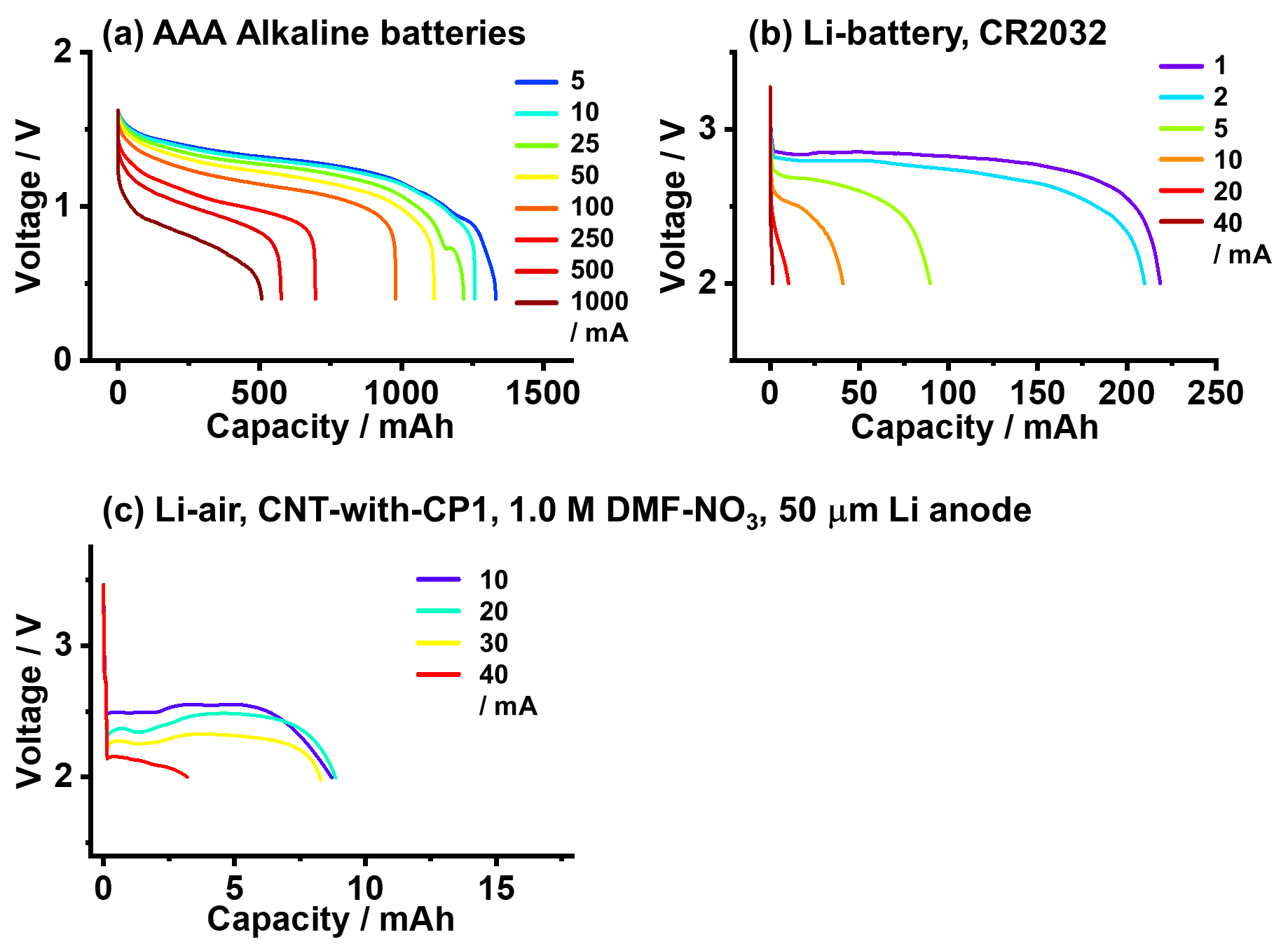


**Figure S13.** (a) Discharge curves of a Toshiba AAA alkaline primary battery with a nominal voltage of 1.5 V and approximate mass of 11.0 g. The total mass of the core cell materials is 7.8 g, excluding the 3.2 g of battery packaging case and current collectors. (b) Discharge curves of an FDK CR2032 lithium primary battery with a nominal voltage of 3 V, nominal capacity of 240 mAh, and approximate mass of 3.0 g. The total mass of the core cell materials (active cathode/anode materials, separator, and electrolyte) is 1.3 g, excluding the 1.7 g stainless-steel cell case. (c) Discharge curves of CNT-with-CP1 cathode cells equipped with a 1.0 M *N*,*N*-dimethylformamide dissolving 1.0 M LiNO_3_ (DMF-NO_3_) electrolyte and 50 μm Li foil anode under dry air (Li–air), obtained from ref. [1]. The total mass of the cell materials is 44.7 mg. The side numbers in the graphs indicate the applied currents in mA.

**Table S2.** Specifications of the reported LAB cell devices tested under atmospheric O_2_ (Li-air). The numbers in parentheses indicate the core cell materials weight excluding the weight of the current collector and packaging materials, and the energy/power density values based on the core cell materials weight.

| Cell No. | Capacity / mAh | Current / mA | Weight / g | Energy density / Wh kg^-1^ | Power density / W kg^-1^ | Reference |
| --- | --- | --- | --- | --- | --- | --- |
| 1 | 1570 | 100 | 5.2  (4.7) | 740  (830) | 48  (53) | This work |
| 2 | 816 | 1.5 | 3.7  (2.9) | 590  (760) | 1.1  (1.4) | [2] |
| 3 | 329 | 1.2 | 1.2  (0.87) | 720  (1000) | 2.6  (3.7) | [3] |
| 4 | 612 | 3.2 | 1.0  (0.95) | 1600  (1600) | 8.2  (8.6) | [4] |

**(Cell No. 1)** Ten-layer stacked cell with double-sided CNT-with-CP1 cathodes in a 2 × 5 cm^2^ dimension. The discharge curve is shown in Figure 8(c), deriving 1570 mAh (3860 mWh) discharge capacity at a current of 100 mA by a 5.2 g cell device (4.7 g excluding current collecting materials weight).

**(Cell No. 2)** Li et al. reported an Li-air pouch cell equipped with a gel polymer electrolyte, demonstrating 816 mAh capacity (2189 mWh) discharge under ambient air. [2] The applied current (1.5 mA) was derived from the reported current density (0.05 mA cm^-2^) and active electrode area (30 cm^2^). The weight of the full pouch cell was 3.7 g, and the total weight of core cell materials (lithium, cathode, separator, and electrolyte) was 2.9 g.

**(Cell No. 3)** Fang et al. reported an Li-air pouch cell equipped with a composite polymer electrolyte with garnet inorganic solid electrolyte, demonstrating 329 mAh capacity discharge at a current of 1.2 mA under ambient air, reporting 720.9 Wh kg^-1^ energy density by a 1.2 g pouch cell. [3] The core cell materials weight was 0.87 g, by deducting 0.06 g current collecting tabs and 0.29 g package weight.

**(Cell No. 4)** Yu et al. reported an Li-air pouch cell equipped with Ce-Mo oxides cathode, demonstrating 612 mAh capacity discharge at a current of 3.2 mA under ambient air, reporting 1560 Wh kg^-1^ energy density by a 1.0 g pouch cell. [4] The core cell materials weight was 0.95 g, by excluding 0.05 g current collector.


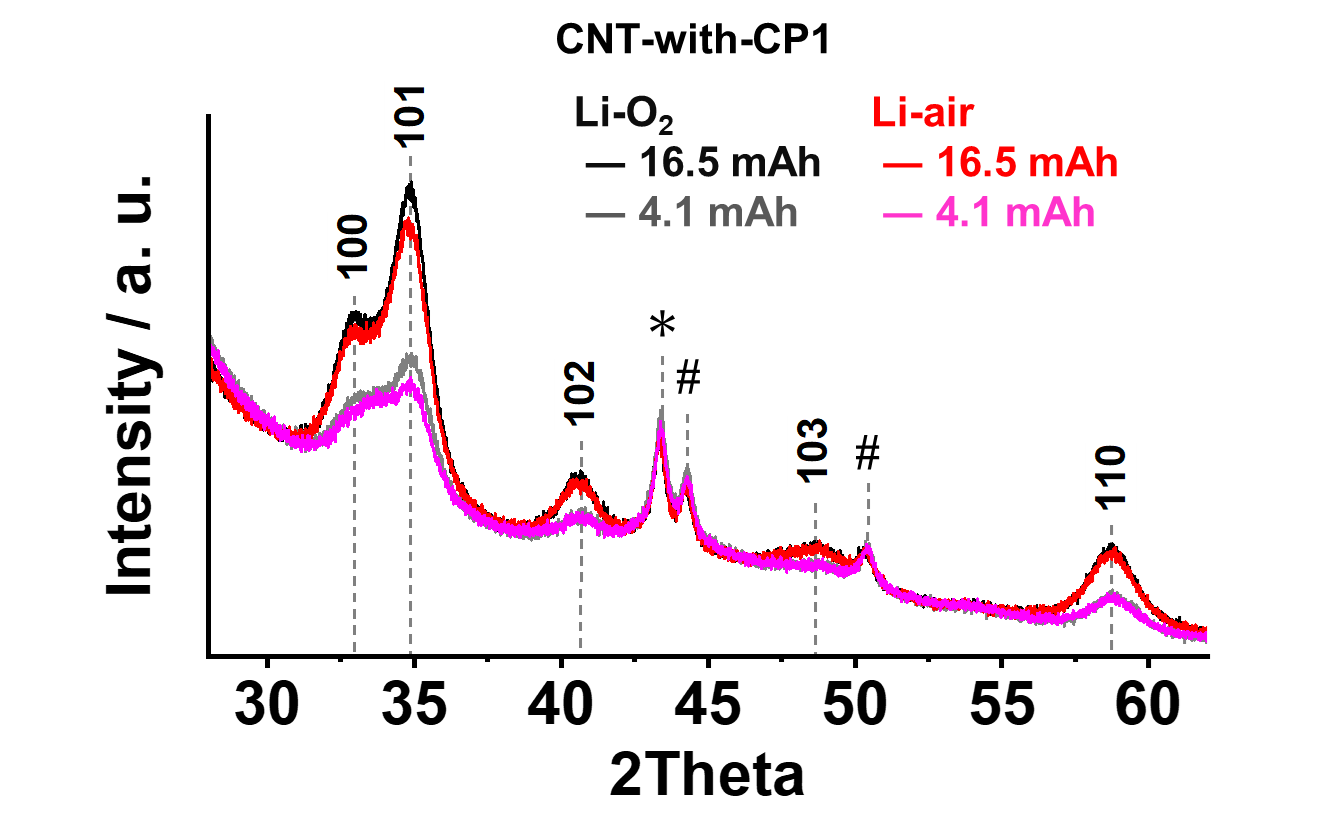


**Figure S14.** XRD spectra of CNT-with-CP1 cathodes after 16.5 mAh and 4.1 mAh discharge at 0.8 mA under Li-O_2_ and Li-air conditions. The numbers in the graph indicate the miller indices for the Li_2_O_2_ crystal diffraction. The * and # symbols denote the reflections from a stainless-steel plate sample holder and CP carbon fiber, respectively.


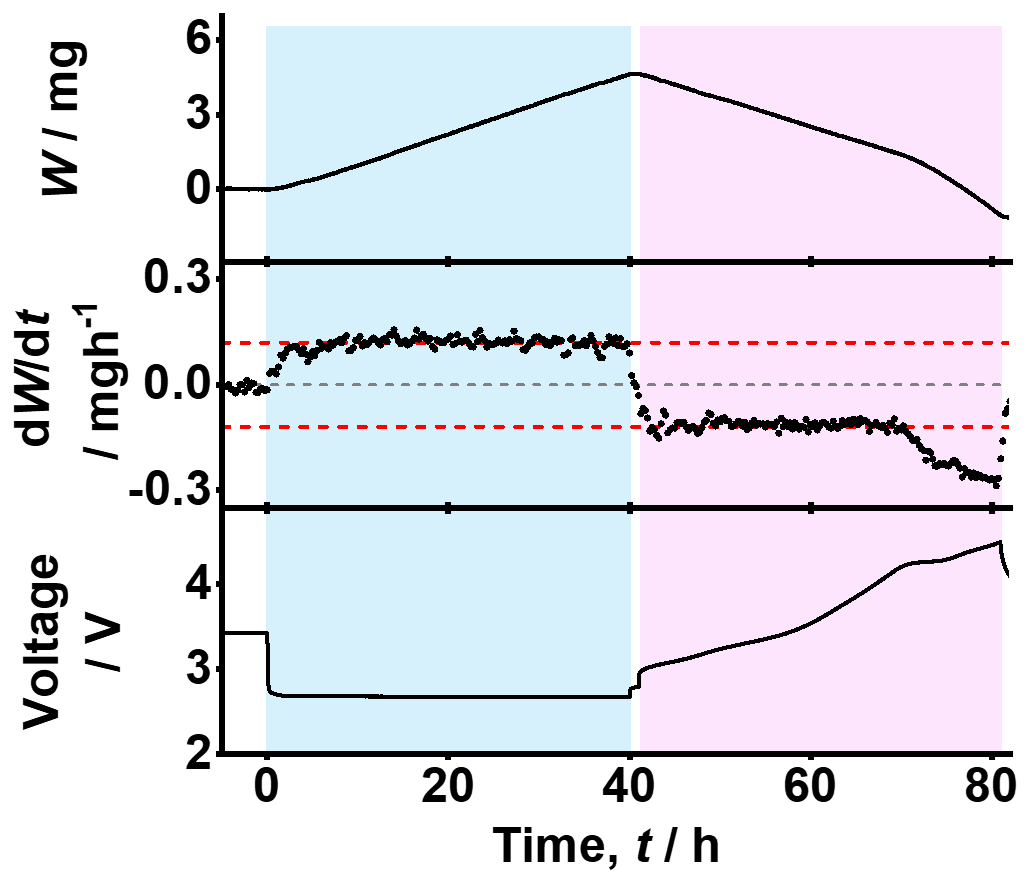


**Figure S15.** Weight change, *W* (up), and its time derivative, d*W*/d*t* (middle), profiles along with the voltage curve (bottom) for an LAB cell with CNT-with-CP1 cathode under Li-O_2_ condition at 0.2 mA × 40 h discharge and charge. The pale blue and red boxes show the regions of discharge and charge, respectively. The red dotted lines in d*W*/d*t* profile represent weight change ratio according to a 2e^-^/O_2_ process at ±0.2 mA (±0.12 mg h^-1^).


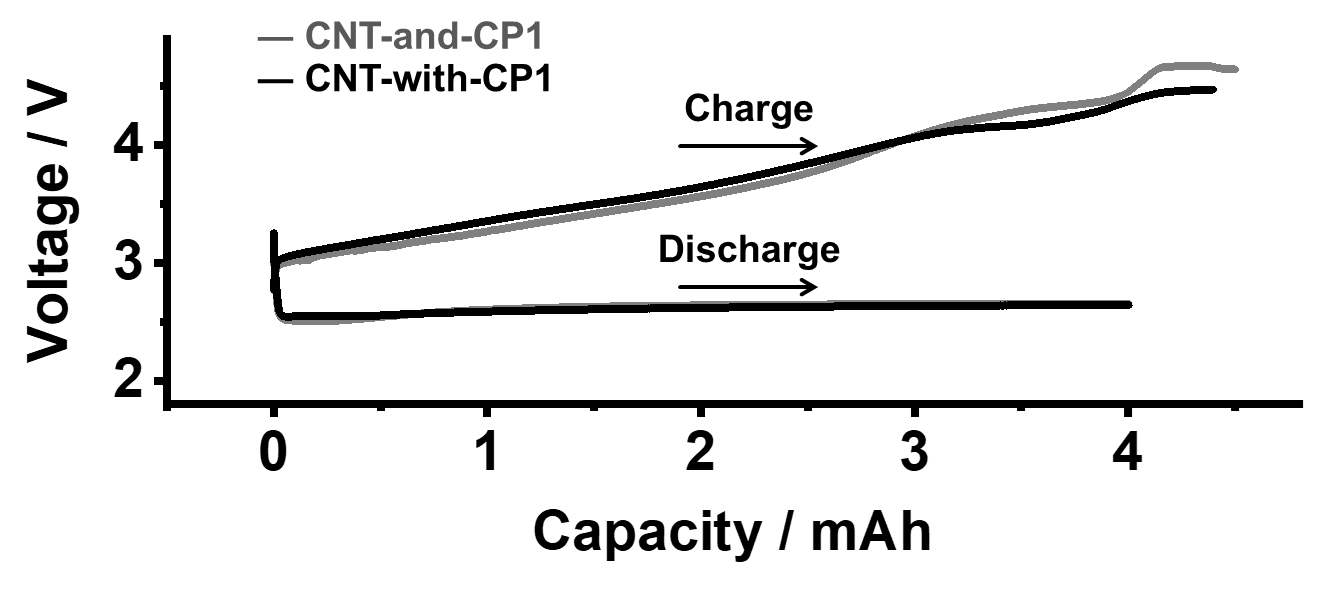


**Figure S16.** Discharge (0.8 mA × 5 h) and charge (0.8 mA × 5.5 h) profiles of CNT-and-CP1 (gray) and CNT-with-CP1 (black) cells in Li–O_2_. A pure He flow was applied while in charging to analyze the gas evolution.


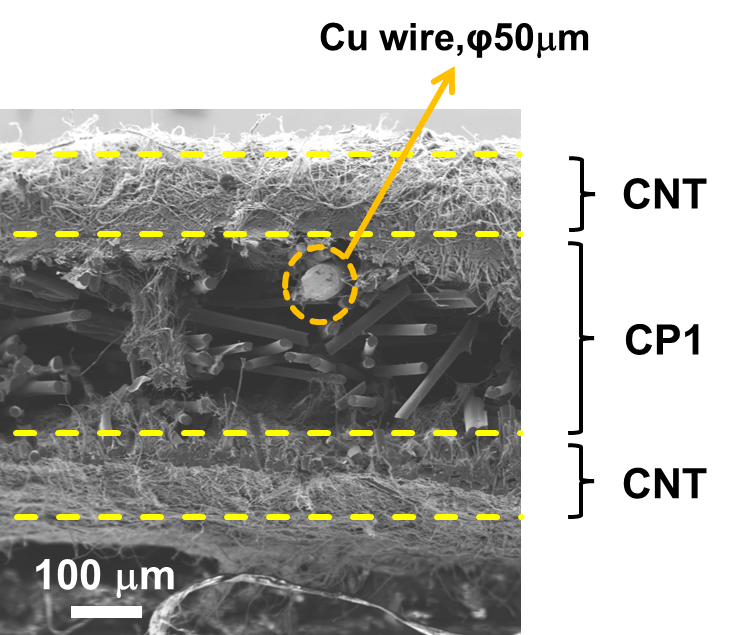


**Figure S17.** SEM image of the double-sided CNT-with-CP1 cross section. The dashed yellow lines indicate the approximate boundaries of the CP/CNT/CP layers. The orange circle indicates the cross section of the Cu wire embedded in the CP1 layer to collect the cathode current.


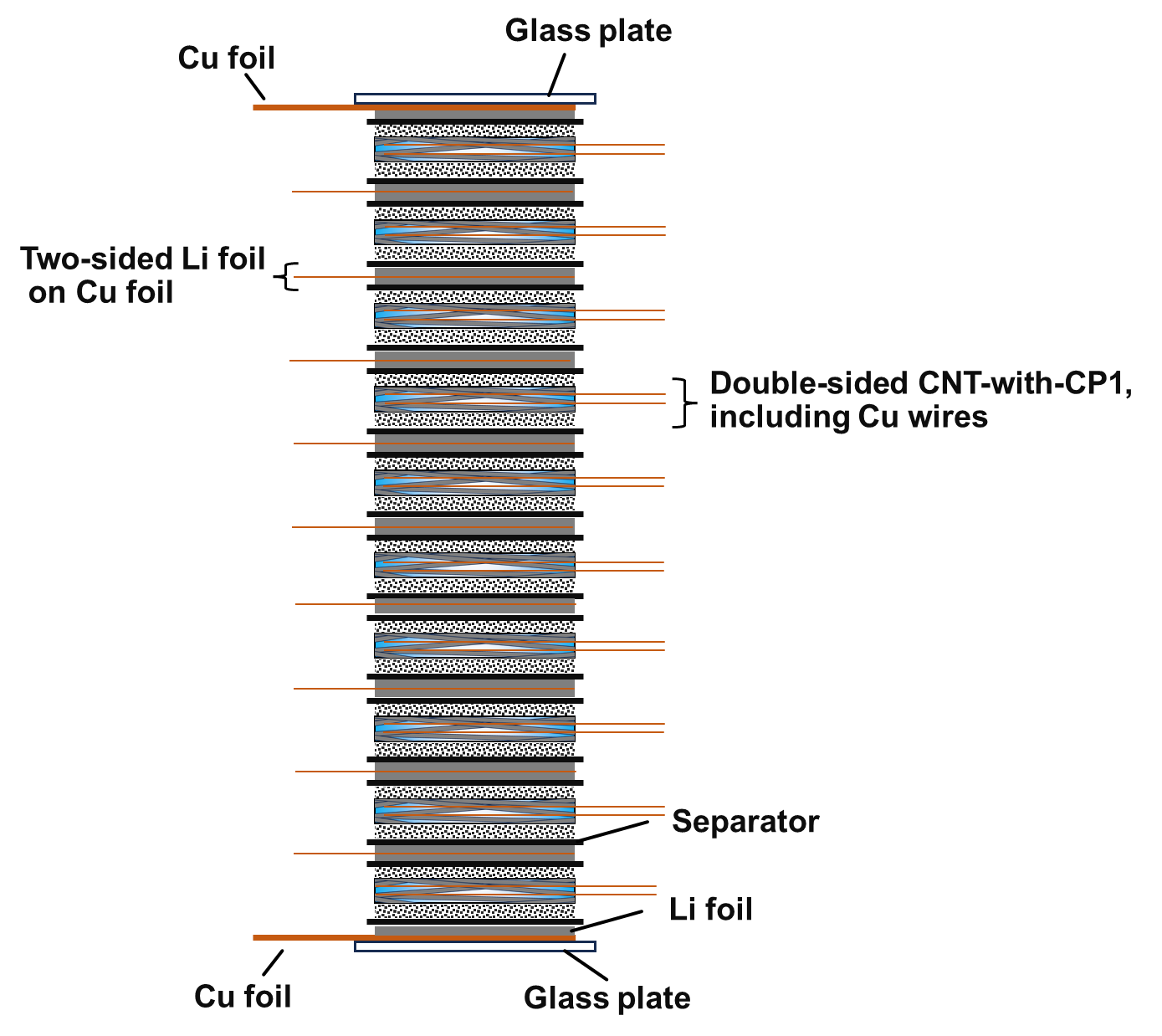


**Figure S18.** Schematic of the ten-layer stacked Li–air cell structure.

**Table S3.** Specifications of the cell materials used for the ten-layer stacked Li–air cell in Figures 8(b) and S12. The numbers in parentheses indicate the weight excluding the weight of the copper foil and copper wire used as current collectors.

| Items | | Unit weight / g | Quantity | Total weight / g |
| --- | --- | --- | --- | --- |
| Li anode | Single 75 μm thick Li foil,  2 × 5 cm^2^ dimension | 0.040 | 2 | 0.08 |
|  | Double-sided 75 μm thick Li foil,  on 6 μm thick Cu,  2 × 5 cm^2^ dimension,  including 0.2 × 2 cm^2^ Cu foil tab | 0.136  (0.080) | 9 | 1.22  (0.72) |
| Separator | Porous poly olefin separator, 20 μm thick,  2.4 × 5.4 cm^2^ dimension | 0.013 | 20 | 0.27 |
| Cathode | Double-sided CNT-with-CP1 cathode,  2 × 5 cm^2^ dimension,  including 50 μm thick 6 cm long two Cu wires | 0.122  (0.120) | 10 | 1.22  (1.20) |
| Electrolyte | | 0.240 | 10 | 2.40 |
| Total | | | | 5.19  (4.67) |

**References**

[1] Nomura A, Azuma S, Ozawa F, et al. Rational Engineering of Amide-Based Electrolytes for “True”-Lithium–Air Batteries Working by Atmospheric Oxygen. ACS Applied Energy Materials 2026. doi:10.1021/acsaem.5c02972

[2] Li Z-W, Liang Y-L, Wang J, et al. An In Situ Gelled Polymer Electrolyte to Stabilize Lithium–Air Batteries. Advanced Energy Materials. 2024;14(19):2304463. doi: 10.1002/aenm.202304463

[3] Fang Q, Sun Y, Cheng J, et al. In situ composite polymer electrolytes for 720 Wh kg⁻¹ and safe Li-air batteries. Energy Storage Materials. 2026;84:104791. doi: 10.1016/j.ensm.2025.104791

[4] Yu H, Zhang G, Yang Q, et al. Orbital Charge Exchange Transition Conceptually Activates Multi-Bifunctional Li–O_2_ and Li–CO_2_ Pathways at Deep and Shallow Energy Levels in Li–Air Batteries. Angewandte Chemie International Edition. e22879. doi: 10.1002/anie.202522879
